# Timing and causes of North African wet phases during the last glacial period and implications for modern human migration

**DOI:** 10.1038/srep36367

**Published:** 2016-11-03

**Authors:** Dirk L. Hoffmann, Mike Rogerson, Christoph Spötl, Marc Luetscher, Derek Vance, Anne H. Osborne, Nuri M. Fello, Gina E. Moseley

**Affiliations:** 1 Max Planck Institute for Evolutionary Anthropology, Department of Human Evolution, Deutscher Platz 6, 04103 Leipzig, Germany; 2 Bristol Isotope Group, School of Geographical Sciences, University of Bristol, University Rd, Bristol, BS8 1SS, UK; 3 Department of Geography, Environment and Earth Sciences, University of Hull, Cottingham Road, Hull, HU6 7RX, UK; 4 Institute of Geology, University of Innsbruck, Innrain 52, 6020 Innsbruck, Austria; 5 Austrian Academy of Sciences, IGF, Technikerstr. 21a, 6020 Innsbruck, Austria; 6 Institute for Mineralogy and Petrology, Department of Earth Sciences, ETH Zürich, Clausiusstr. 25, 8092 Zürich, Switzerland; 7 GEOMAR Helmholtz Centre for Ocean Research Kiel, Wischhofstraße 1-3, 24148 Kiel, Germany; 8 National Oil Corporation (NOC), Exploration Department, P. O. Box 2655, Tripoli, Libya

## Abstract

We present the first speleothem-derived central North Africa rainfall record for the last glacial period. The record reveals three main wet periods at 65-61 ka, 52.5-50.5 ka and 37.5-33 ka that lead obliquity maxima and precession minima. We find additional minor wet episodes that are synchronous with Greenland interstadials. Our results demonstrate that sub-tropical hydrology is forced by both orbital cyclicity and North Atlantic moisture sources. The record shows that after the end of a Saharan wet phase around 70 ka ago, North Africa continued to intermittently receive substantially more rainfall than today, resulting in favourable environmental conditions for modern human expansion. The encounter and subsequent mixture of Neanderthals and modern humans – which, on genetic evidence, is considered to have occurred between 60 and 50 ka – occurred synchronously with the wet phase between 52.5 and 50.5 ka. Based on genetic evidence the dispersal of modern humans into Eurasia started less than 55 ka ago. This may have been initiated by dry conditions that prevailed in North Africa after 50.5 ka. The timing of a migration reversal of modern humans from Eurasia into North Africa is suggested to be coincident with the wet period between 37.5 and 33 ka.

The North African region experienced dramatic changes in climatic and hydrological conditions during the Pleistocene^1^ with important consequences for human evolution and dispersal. The region lies at a critical boundary position between polar and subtropical air masses and its climate is influenced by both the Mediterranean Sea and the Atlantic Ocean. However, the specific mechanisms behind these influences and resulting hydrological changes are not well understood. Marine records of dust and runoff^2^,^3^ as well as Holocene terrestrial records^4^,^5^, point to the over-riding importance of the precession cycle in controlling North African rainfall^6^. On the other hand, there is a lack of terrestrial evidence for past precipitation, particularly for the pre-Holocene for which well-dated time series are almost absent.

Speleothems, especially stalagmites, are well established terrestrial archives of past environmental and climate conditions through time series of proxies including δ^18^O, δ^13^C and trace element concentrations^7^. Under specific circumstances, speleothem formation itself represents a direct proxy for hydrological changes at sites where water availability was highly variable in the past. For example, the very presence of speleothems in caves located in currently arid areas indicates dramatic changes in water availability and epikarst recharge^8^,^9^. We present a palaeohydrological record based on a stalagmite from Susah Cave (Fig. 1, 32°53.419′N, 21°52.485′E), a shallow cave positioned at ~200 m altitude on the northern flank of Jebel Malh in the Al Akhdar Massif in Cyrenaica, Libya, in close proximity to Haua Fteah, an archaeological cave site with evidence for human occupation since about 75 ka^10^,^11^. Today, the region experiences average precipitation of less than 200 mm per year, with most rainfall between October and April, sustaining a Mediterranean ‘Maquis’ vegetation with a thin soil cover. Reflecting this low rainfall, Susah Cave is hydrologically inactive today, with no evidence of water flow or dripping anywhere in the cave. Speleothem formations are covered with dust, and the majority of the specimens are broken. The sample used here (SC-06-01) is a 93 cm-long stalagmite that was found broken, but the basal piece fitted onto its stump so that completeness of the specimen could be demonstrated (Supplementary Information (SI), Fig. S2). Thin red layers similar to the superficial dust coating separate individual growth intervals, and mark episodic dry phases or events with concomitant dust deposition inside the cave. The chronology and derived age model are based on 116 reliable and precise U-Th dates^12^ along the growth axis (SI, Table S1). For palaeoclimatic analyses, stable oxygen and carbon isotopes (δ^18^O and δ^13^C) were measured along the growth axis at 1 mm resolution or, for sections with slow growth, at 0.15 mm.

## Growth phases and insolation

The presence of speleothems in Susah Cave demonstrates that effective rainfall must have been locally significantly higher in the past, allowing for episodic groundwater recharge in coastal northeast Libya. SC-06-01 formed episodically during the last glacial period between 67 and 30 ka (Fig. 2). We identify three sustained speleothem growth phases (Fig. 2, phases I, II and III) and 12 minor growth phases (phases 1 to 12) during the last glacial period (SI, Figs S3 and S4) during which increased aquifer recharge led to speleothem formation in Susah Cave. The three main sustained growth phases represent about 75% of stalagmite deposition. The SC-06-01 δ^13^C_calcite_ record (SI, Fig. S8) supports the interpretation that the sustained growth phases I, II and III represent times of higher water availability and hence North African wet phases. At times of more water availability the vegetation density increases and leads to higher pCO_2_ which in turn leads to increased carbonate dissolution in the epikarst. Consequently, phases I, II and III exhibit higher growth rates. During phases I, II and III we find lower δ^13^C_calcite_ values, while the short growth phases, particularly between 50 and 40 ka, are characterised by higher δ^13^C_calcite_ values. These compositions likely result from changes in vegetation density, C4 vs. C3 vegetation, or prior calcite precipitation. At times of less water availability a relative increase of C4 vegetation would yield higher δ^13^C_calcite_ values. Lower vegetation density, also a result of less water availability, likewise leads to higher δ^13^C_calcite_ values. And increased prior calcite precipitation as a results of drier conditions also gives rise to higher δ^13^C_calcite_ values. All this strongly indicates that the main growth phases I, II and III represent major North African wet phases. Two of the phases during which the speleothem grew rapidly, SC-06-01 phase I (65 to 61 ka) and phase III (37.5 to 33.5 ka), occur ~3000 years before maxima in Northern Hemisphere summer insolation^13^, arising from precession minima (Fig. 3). This supports a precessional forcing of runoff^14^ and freshwater supply to the Mediterranean Sea^6^, as well as reduced aeolian dust supply to both the Mediterranean^15^ and the eastern Atlantic^16^. Rainfall changes in North Africa and the surrounding region have previously been ascribed to meridional migrations of the Intertropical Convergence Zone (ITCZ) and its attendant monsoon belt^14^,^17^. Cyrenaica is too far north to be directly influenced by the monsoon, but the coherence of variability in SC-06-01 and the precessional control at low latitudes suggests a teleconnection. Most likely, rainfall in Cyrenaica reflects enhanced convergence at 25–40°N as a consequence of the contraction of the Hadley cell, as suggested by General Circulation Model (GCM) experiments^18^ and proxy data^19^.

A shorter main phase of sustained speleothem growth, phase II (52.5 to 50.5 ka), is not related to a precession minimum, and therefore reflects a different component of the climate system. SC-06-01 phase II occurs ~ 3000 a before a maximum in obliquity (Fig. 3). This is the first terrestrial archive-based support of the model-based hypothesis^18^ that North African precipitation is enhanced during both maximum obliquity and minimum precession. Indeed, according to GCM results the impact of obliquity on insolation should be stronger during maximum precession^18^, and this is consistent with what we observe in SC-06-01 phase II.

A comparison between speleothem growth phases and insolation at 30°N and 60°N (SI, Figs S11 and S12) indicates that increased North African precipitation during the last glacial period could have been caused by the insolation gradient between mid and high latitudes. Phases I and III occurred just before peaks of the gradient in winter insolation between 60°N and 30°N (SI, Fig. S11b), whilst phase II occurred before a peak of gradient in summer insolation (SI, Fig. S12b). This mixed forcing indicates that increased water supply to North Africa can be the consequence of more than one configuration of insolation, in a way that is perhaps analogous to today’s North Atlantic Oscillation (NAO). Increasing insolation at 60°N relative to 30°N (SI, Figs S11 and S12) could more permanently reduce the gradient between the Icelandic Low and the Azores High, leading to a prolonged NAO^−^-like scenario, with wet climate in southern Europe and northern Africa while dry and cold conditions prevail in central and northern Europe^20^. This idea receives support from the fact that discontinuities in a stalagmite from Villars cave (France)^21^, interpreted as cold and dry phases in Europe, coincide with humid phases recorded by SC-06-01 (Fig. 3). The relationship between winter insolation and NAO^−^-like winter precipitation is straightforward during phases I and III, but a less direct mechanism must connect summer insolation and NAO^−^-like conditions during phase II. This may reflect the fact that winter precipitation in North Africa is largely driven by the North Atlantic surface heat budget^22^, which is influenced by both winter and summer insolation.

A key new observation in the SC-06-01 record is that all three main phases of speleothem growth occur before the corresponding orbital peak, and so rainfall occurs during increasing obliquity or decreasing precession, and not at maximum or minimum values respectively. The timing of growth phases found in SC-06-01 is precise to less than 300 a (2σ). The wet phases are out of phase with peak precession and obliquity by ~3000 a (Fig. 3). The observation that coastal North African rainfall precedes precession minima by ~3000 a is inconsistent with the accepted phase relationship between runoff from North Africa and precession, inferred from the timing of sapropel formation in the eastern Mediterranean^6^,^23^. Based on the radiocarbon age control on the Holocene Sapropel 1, and astrochronological arguments for the Neogene^6^, peak monsoonal runoff has been considered to lag peak precession minima by ~3000 a. Coastal rainfall is therefore out of phase with the monsoonal rainfall responsible for sapropel formation, which could also explain the shift in timing of Atlantic dust minima^16^ relative to that of sapropels. It seems likely that during the first millennia of the African Humid Periods, dust transport was suppressed by northern rainfall as recorded in SC-06-01. During the later millennia of these humid periods, rainfall further south suppressed dust transport and enhanced run off, which eventually led to sapropel deposition.

## Growth phases and Greenland Interstadials

Growth phases in Susah Cave are not only controlled by orbital insolation. A succession of minor growth episodes of SC-06-01 mark millennial changes in water availability. Most of these minor episodes (phases 2, 3b, 4, 6, 7, 8, 9, 10 and 11) coincided with Greenland Interstadials (GIS) (Fig. 3) as recorded in the NGRIP δ^18^O record^24^ (although they are numbered differently), indicating increased effective rainfall in northern Africa during times of rapid Northern Hemisphere warming. This is consistent with a marine MIS 3 record from the Western Mediterranean Sea, which shows stadials with lower sea-surface temperatures and higher aridity, and interstadials with higher continental humidity^25^. However, some of the minor growth phases in SC-06-01 (1, 3a, 5 and 12) coincide with cold periods in Greenland. This phenomenon has not previously been recognised for North African rainfall, and further investigation will require additional records from the region with age control as good as SC-06-01.

**Figure 1:**
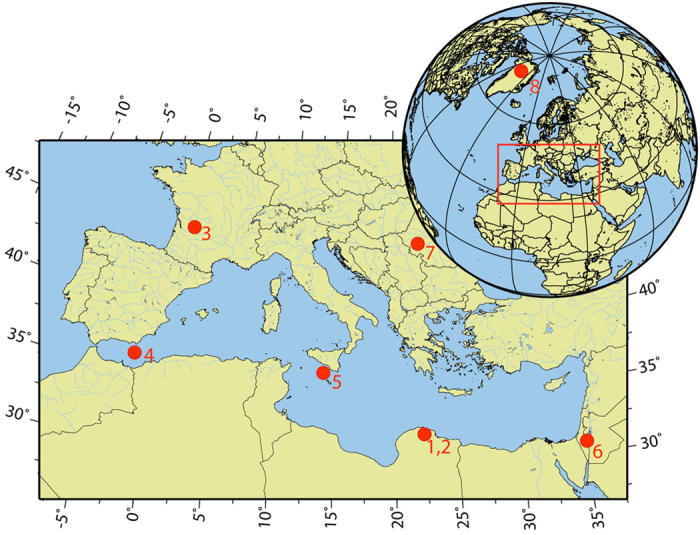
Map showing the locations of 1, 2) Susah Cave in northern Libya and nearby site Haua Fteah^11^, 3) Villars Cave^21^ in France, 4) marine sediment core ODP 977^28^, 5) marine sediment core ODP 963^27^, 6) Levant with archaeological sites Kebara^44^ and Boker Tachit^39^, 7) Peştera cu Oase^48^ and 8) NGRIP ice core^24^ . The map was created using the Geomar GMT-Maps web site (http://sfb574.geomar.de/gmt-maps.html).

**Figure 2:**
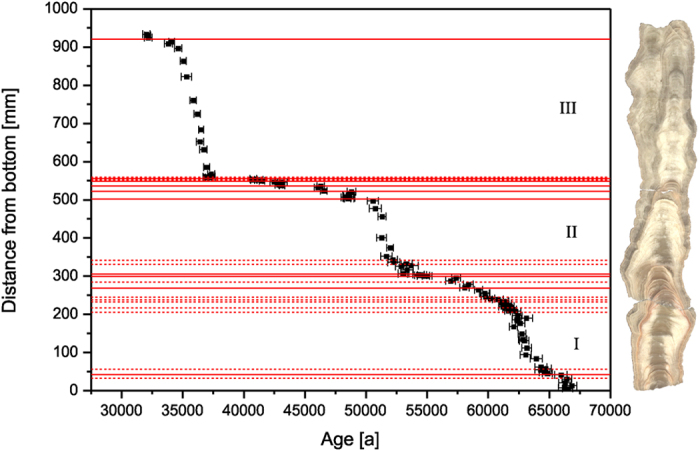
Chronology of SC-06-01. U-Th ages are shown vs distance from the bottom of the stalagmite. The polished sections of the stalagmite are shown on the right for comparison. Red lines indicate positions of red dust layers visible in the speleothem. Solid red lines represent red layers associated with a hiatus, red layers without a detectable hiatus within dating uncertainties are marked by dotted red lines. The three main growth phases are the sections I, II and III.

**Figure 3:**
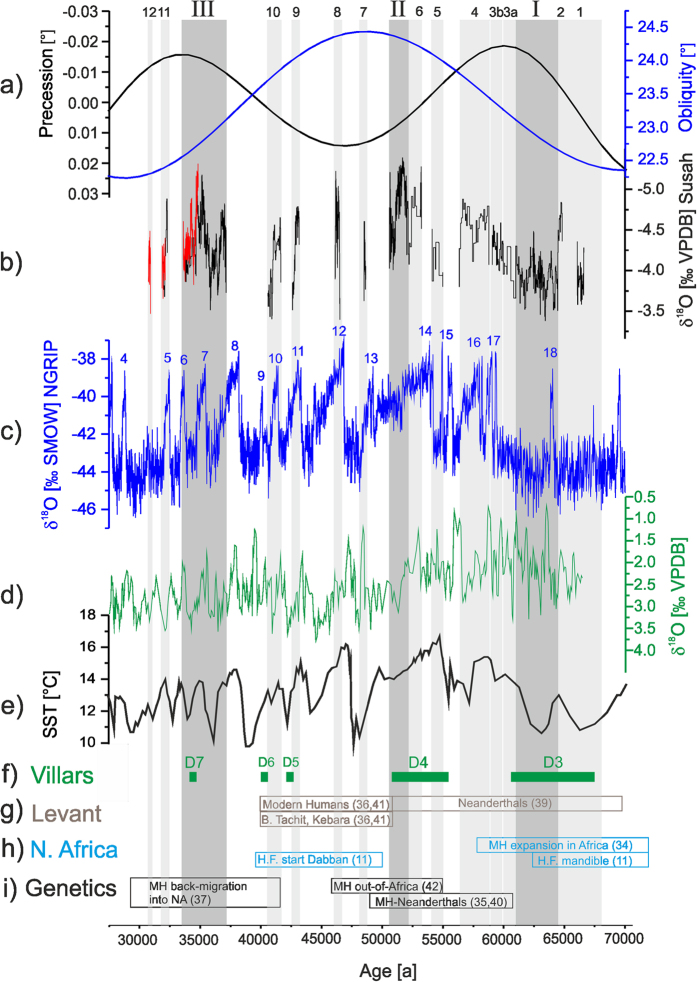
Speleothem δ^18^O_calcite_ record (**b**) from northern Libya (Susah), numbers on top of the grey bars indicate SC-06-01 growth phases. The red data points of the SC-06-01 δ^18^O record refer to the twin stalagmite near the top. Dark grey bars indicate main phases I, II and III of sustained growth and light grey bars indicate phases 1 to 12 of short growth intervals. Comparison of the δ^18^O_calcite_ with (**a**) Northern Hemisphere obliquity (blue) and precession (black) changes^13^, (**c**) NGRIP δ^18^O^24^ (blue numbers on top of the NGRIP record indicate Greenland Interstadials), (**d**) Mediterranean planktonic δ^18^O^27^, (**e**) Mediterranean SST^28^, (**f**) timing of discontinuities (green bars) in Villars cave speleothem^21^, durations of D5, D6 and D7 are not resolved by the age model of Vil9, (**g**,**h**) timing of archaeological evidence in North Africa and the Levant, (**i**) timing of genetically derived events in human evolution. Note that the scale is reversed for precession, the Mediterranean δ^18^O and the SC-06-01 δ^18^O record.

Changes in δ^18^O in speleothem calcite can be driven by a variety of effects^7^, for example, by changes of the rainwater source, moisture trajectories, ocean surface water composition (ice volume), amount effect, air temperature, and local effects in the cave such as drip rate, pCO_2_ or cave air temperature^26^. The position of Susah cave (Fig. 1) suggests that the main precipitation source is the Mediterranean Sea, but Atlantic influences are also likely. In Fig. 3 the SC-06-01 δ^18^O record is compared to δ^18^O records from Mediterranean δ^18^O_sea_^27^ and NGRIP δ^18^O_ice_^24^ as well as Mediterranean sea-surface temperature (SST)^28^. δ^18^O_sea_ and SST show coherent patterns, consistent with a Mediterranean source for at least some rainfall at Susah cave. Changes in global ice volume between 65 and 30 ka may be an additional factor controlling the absolute value of δ^18^O in SC-06-01 on timescales ~10 ka, but this is probably not first-order effect on the high-frequency δ^18^O_calcite_ changes recorded by SC-06-01 since ice volume-related source changes are not known on Dansgaard-Oeschger frequencies. However, comparison of the SC-06-01 δ^18^O_calcite_ record with δ^18^O_ice_ from NGRIP also indicates a high degree of correspondence, and the two records appear to be in-phase (Fig. 3 and SI, Fig. S9 and S10). This is true for both precessional and millennial timescales, regardless of the phasing with interstadials/stadials. Part of the SC-06-01 record is duplicated along a parallel secondary growth axis (Fig. 3), and this partial replication confirms the isotope pattern of SC-06-01. The correlation of the SC-06-01 and NGRIP data series indicates a North Atlantic influence on rainfall composition in North Africa, with warm periods in Greenland corresponding to isotopically more negative rainfall in Libya. This does not seem to arise from the isotopic ‘amount effect’ as found for speleothems in the Levant^29^, although some influence of the rainfall amount on the δ^18^O record cannot be excluded. For SC-06-01, maximum speleothem growth likely reflects maximum water availability, but the main growth phases I and II in our speleothem correspond to both higher (-3.8‰ between 65 and 61 ka) and lower δ^18^O values (-5.3‰ between 52.5 and 50.5 ka). This means that there are independent controls on rainfall amount and water isotopic composition. Similarity between relative changes in the North Atlantic system (NGRIP) and δ^18^O_calcite_ therefore indicate a partial Atlantic source of the rainfall recorded in SC-06-01, perhaps via the frequency and severity of Atlantic storms influenced by the Atlantic SST. An Atlantic source is also implied by the δ^18^O_calcite_ values, which are low relative to expectations for the Mediterranean region at this time^30^. We therefore interpret the isotopic variability in the SC-06-01 record as indicating that warm phases in Greenland correspond to increased advection of Atlantic-sourced moisture to Libya. This interpretation is consistent with GCM results, which show enhanced advection of moisture from the Atlantic across coastal North Africa during Northern Hemisphere warm phases^22^,^31^.

## Implications for modern human dispersal and migration

Our speleothem record not only reveals important insights into North African hydrology changes during the last glacial period, but also sheds light on the highly variable environmental boundary conditions for human migration and dispersal during this critical time period. North Africa has been suggested to be a potential ‘launch pad’ for modern human migration into Eurasia^32^, and advantageous hydroclimate conditions, as identified in our record, are the primary limitation on the ability of the North African region to play this role. Migration of modern humans out of the sub-Sahara region into the North African realm would have been possible during MIS 5 due to a Sahara wet phase resulting in a green Sahara scenario^33^ and connected river systems^34^,^35^. However, subsequent migration and/or dispersal patterns north of the Sahara between 70 and 45 ka are still unclear. Our record shows that northern Africa remained humid during most of MIS 4 after the “green Sahara” phase ended about 72 ka ago. We show that North Africa experienced a sustained wet phase with favourable conditions for humans at 65–61 ka (SC phase I), a time when the Saharan area was dry^36^, but modern human populations show a major expansion in Africa^37^. Modern human remains have been found in Haua Fteah cave, only a few kilometres from Susah Cave, in a layer dated to about 73–65 ka^11^. Our results suggest that modern human presence and expansion in North Africa can be assigned to the SC phase I wet period recorded in our stalagmite.

The SC-06-01 palaeohumidity record furthermore provides a chronological and palaeoenvironmental framework for at least three subsequent major events in modern human migration and dispersal: a) interbreeding of modern humans with Neanderthals^38^, b) migration of modern humans into Eurasia^39^ and c) back-migration of a non-African lineage into North Africa^40^.

Our data suggest that SC wet phase II between 52.5–50.5 ka is a potential period during which modern human and Neanderthal interbreeding was first possible, perhaps in the Levant. There is independent evidence for humid conditions in the Levant broadly between 60 and 45 ka^41^. This indicates that synchronous humid conditions in North Africa and the Levant prevailed between 52.5 and 50.5 ka possibly along the entire North African coast, providing favourable habitats and migration routes for modern humans. Our results therefore suggest that modern humans expanded into the Levant during SC-06-01 wet phase II between 52.5 and 50.5 ka, where Neanderthals were present after 70 ka^42^. This supports and constrains DNA based timing of Neanderthal - modern human encounter and interbreeding between about 60 and 50 ka^38^,^43^. Archaeological evidence for modern humans in the Levant is found e.g. in Boker Tachit^39^ and Kebara^44^. Although the chronology for these sites is not secure^39^,^45^, modern human presence in the Levant can be broadly assigned to at least around or after 50 ka, while modern humans also remained in North Africa as demonstrated by the start of the Dabban in Haua Fteah^11^.

The further expansion of modern humans into Eurasia was suggested to have started around 48 ka ago^39^. A recent mtDNA based study by Posth *et al.*^46^ indicates a single dispersal out of North Africa starting less than 55 ka ago. Our record provides a possible chronological and environmental framework for this phase of human expansion, too. Dispersal into the Levant was likely to happen during SC wet phase II, possibly followed by the expansion into Eurasia, perhaps during GIS 12^47^ and possibly triggered by the predominantly dry period that started in North Africa after the end of SC wet phase II around 50 ka. Modern humans arrived in Europe no later than 41 ka as evidenced by a mandible found in Peştera cu Oase^48^.

The back-flow of modern humans from Eurasia into North Africa^40^ is another important movement during the last glacial period. Based on mtDNA evidence, Secher *et al.*^40^ show that a non-African lineage was moving back into Africa around 35 ka ago^40^. However, the DNA based estimate for the timing has large uncertainties and ranges between 24.6–46.4 ka. We suggest that this back-flow into the North African realm happened during a North African wet period recorded as SC phase III between 37.5 and 33.5 ka (SC phase III), providing environmental context for this event for the first time.

## Conclusions

SC-06-01 records orbitally forced changes in water availability reflecting low-latitude migration of the ITCZ^17^ as well as high-latitude North Atlantic heat-forced changes in both rainfall amount and composition. This is the first time increased effective rainfall during the last glacial period has been directly demonstrated for the North African region, and extends the spatial distribution of known past increases in effective rainfall in North Africa^5^. This provides new insights for the critically under-constrained northern margin of the African Hadley Cell, and underscores the importance of North Africa as a key region for unravelling teleconnections between low- and high-latitude climate forcing.

Human dispersal and migration in North Africa during the last glacial period were closely linked to water availability. This new record provides important insights into environmental conditions in North Africa, covering the time of modern human dispersal north of the Sahara, interbreeding between Neanderthals and modern humans, modern human migration into Eurasia and back-flow of a non-African lineage into Africa.

## Methods

The chronology of SC-06-01 is based on high-precision MC-ICPMS U-series dating of 116 sub-samples along the growth axis. Sample preparation and analytical protocols are detailed elsewhere^12^. Results are reported in full in the Supplementary Information (SI, Table S1). The depth-age model was derived using StalAge^49^. The dating results show episodic growth and age modelling was accordingly done section-wise using red dust layers as marker points to define growth intervals. Samples for stable isotope analyses were analysed using mass spectrometry as described in Spötl & Vennemann^50^. The isotopic composition of calcite was analysed in eighteen Hendy tests along individual growth layers from the central axis towards the flank of the stalagmite.

## Additional Information

**How to cite this article**: Hoffmann, D. L. *et al.* Timing and causes of North African wet phases during the last glacial period and implications for modern human migration. *Sci. Rep.* **6**, 36367; doi: 10.1038/srep36367 (2016).

**Publisher’s note**: Springer Nature remains neutral with regard to jurisdictional claims in published maps and institutional affiliations.

## Supplementary Material

Supplementary Information
