# Supplementary material for: Timing and causes of North African wet phases during the last glacial period and implications for modern human migration
Source: Sci Rep. 2016 Nov 3;6:36367. doi: 10.1038/srep36367 (PMC5093410; doi:10.1038/srep36367)
Supplement: Supplementary Information [file srep36367-s1.pdf]

Supplemementary information

Timing and causes of North African wet phases during the last glacial period and implications for Modern Human migration

Dirk L. Hoffmann, Mike Rogerson, Christoph Spötl, Marc Luetscher, Derek Vance, Anne H. Osborne, Nuri M. Fello, Gina E. Moseley

Location Susah Cave

Susah Cave (32°53.419' N, 21°52.485' E) is a shallow cave positioned at ~200 m altitude on the northern flank of Jebel Malh in the Jebel Al Akhdar in Cyrenaica (Fig. S1). The cave formed in the Dernah Formation, which consists of hard and massive Eocene limestones, with some dolomitised layers [1]. The cave consists of three large (~10 m radius), roughly circular chambers separated by ~1 m high fossil phreatic tubes. Today, Susah Cave is completely fossil with no evidence of contemporary water flow anywhere in the cave. Formations are covered by dust, and the majority are broken. The region experiences ~180 mm precipitation per year, with most rainfall generally occuring between October and April. A map with recent precipitation patterns of the wider area can, for example, be found in Engelstaedter et al. (2006)[2]. There is only thin soil cover and the vegetation consists of Mediterranean "Maquis".

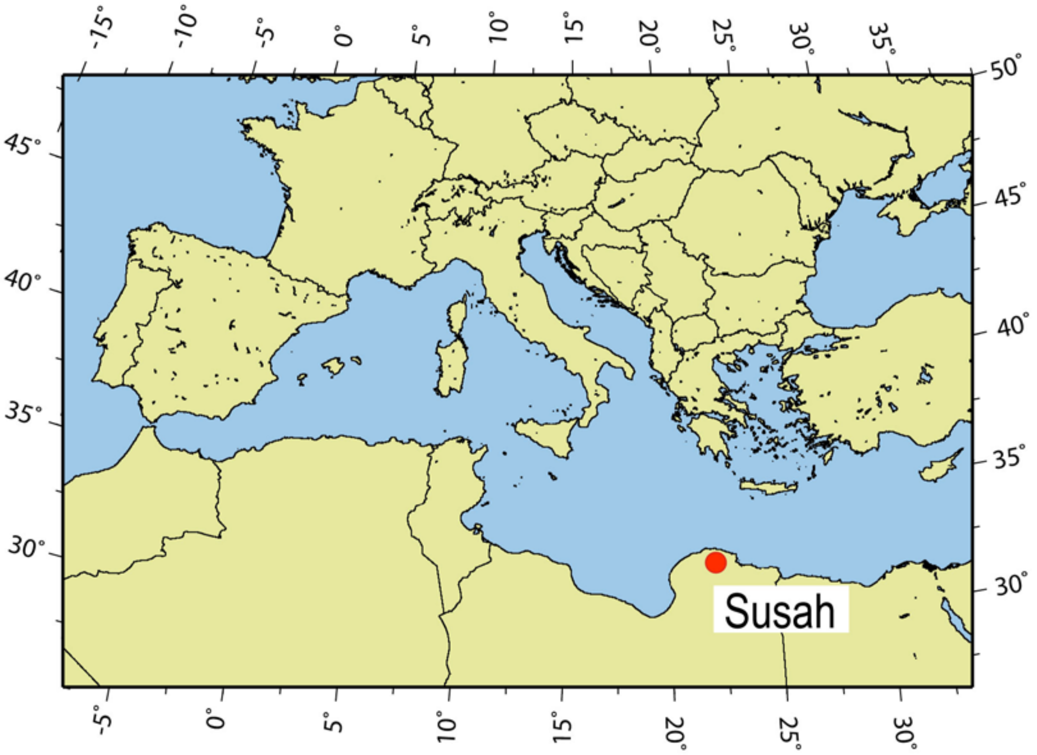

Fig. S1: Map with location of Susah Cave, Cyrenaica (Libya). The map was created using the Geomar GMT-Maps web site (http://sfb574.geomar.de/gmt-maps.html).

Stalagmite SC-06-01

The sample used for this study is a 93 cm-long stalagmite (SC-06-10), which was found broken on site. The three broken pieces fit together and the bottom also fits onto a stump found in the cave. Thus it represents the complete specimen with no missing parts. The diameter varies along the growth axis between 11 and 16 cm. The stalagmite consists of sections of dense clear calcite and milky white calcite. There are several thin red layers similar in colour to the surface dust layer, indicating dust deposition during frequent drying of the stalagmite surface. The main growth axis also shows some slight changes in growth direction. Over the top 30 cm a twin stalagmite formed.

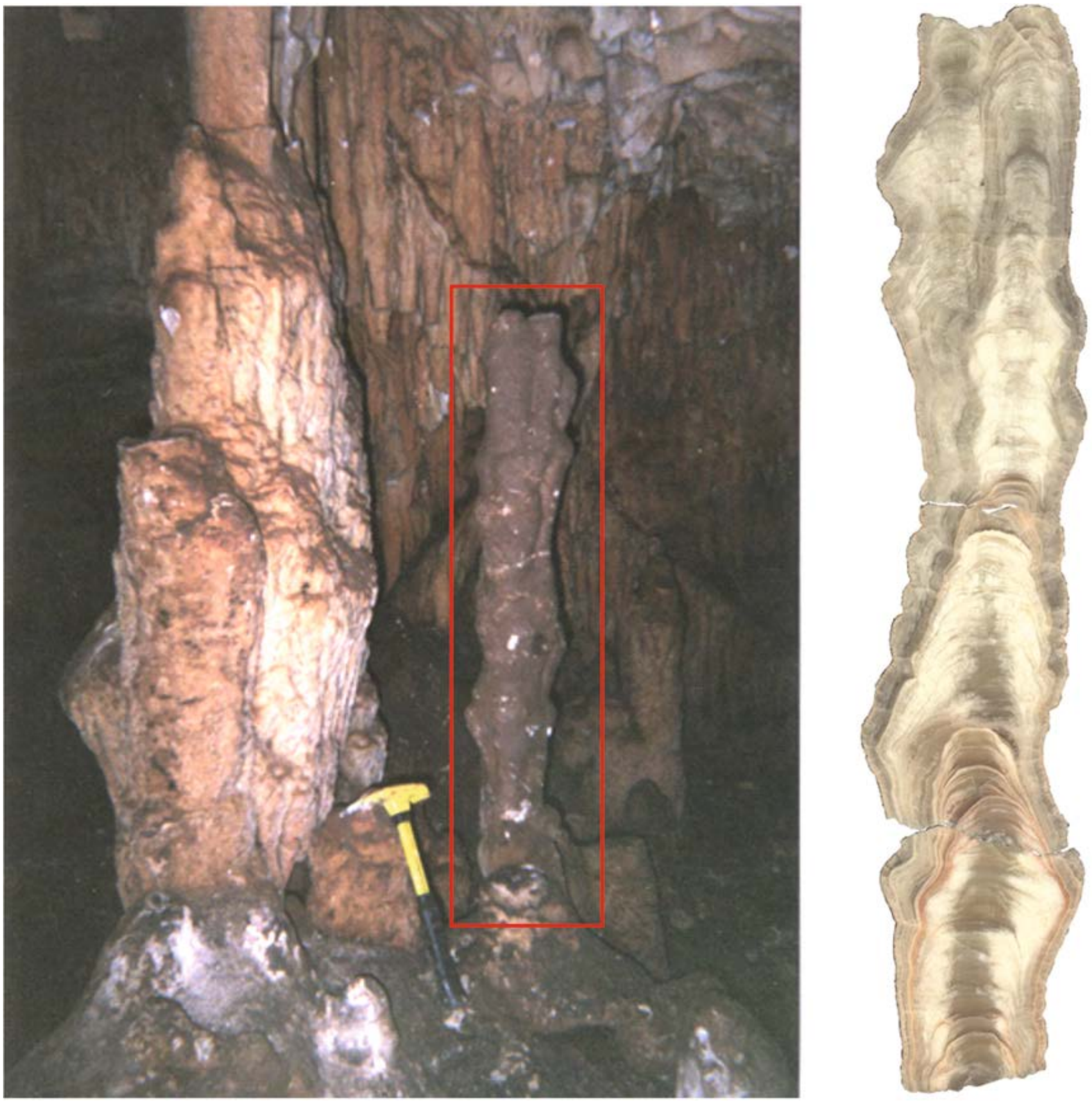

Fig. S2: SC-06-01 (in red box) pieces put together on the stump inside the cave (left) and polished cross section along the growth axis (right).

We defined three main growth sections and twelve minor growth sections based on the crystal structure and visual layering, positions of red layers in the stalagmite and chronological results (see below). Half of the red layers are associated with a hiatus identified by U-series

chronology data and mark the start / end of a growth phase. The other red layers are probably also associated with (short) hiatuses, which cannot be constrained within uncertainty of U-series results. The main sections are all longer than 16 cm, and cover 75 % of the total length of growth without detectable hiatuses. Section I is between 5.6 and 23.7 cm from the bottom, section II from 34.2 to 50.2 cm and section III from 55.4 to 92.1 cm (plus 19 cm of the parallel growth axis). The minor sections are short episodes of stalagmite formation and display frequent red layers indicating dry phases in Susah Cave. Minor sections are a maximum of 4 cm long and together cover 25 % of the stalagmite formation.

Figures S3 a-c show the three polished pieces of SC-06-01. The grey bars indicate the main axis, dark grey represents main sections I, II and III, light grey represents minor sections 1 to 12. The red lines indicate the positions of red coloured layers. Dotted layers are not associated with a longer hiatus exceeding uncertainties in the dating results.

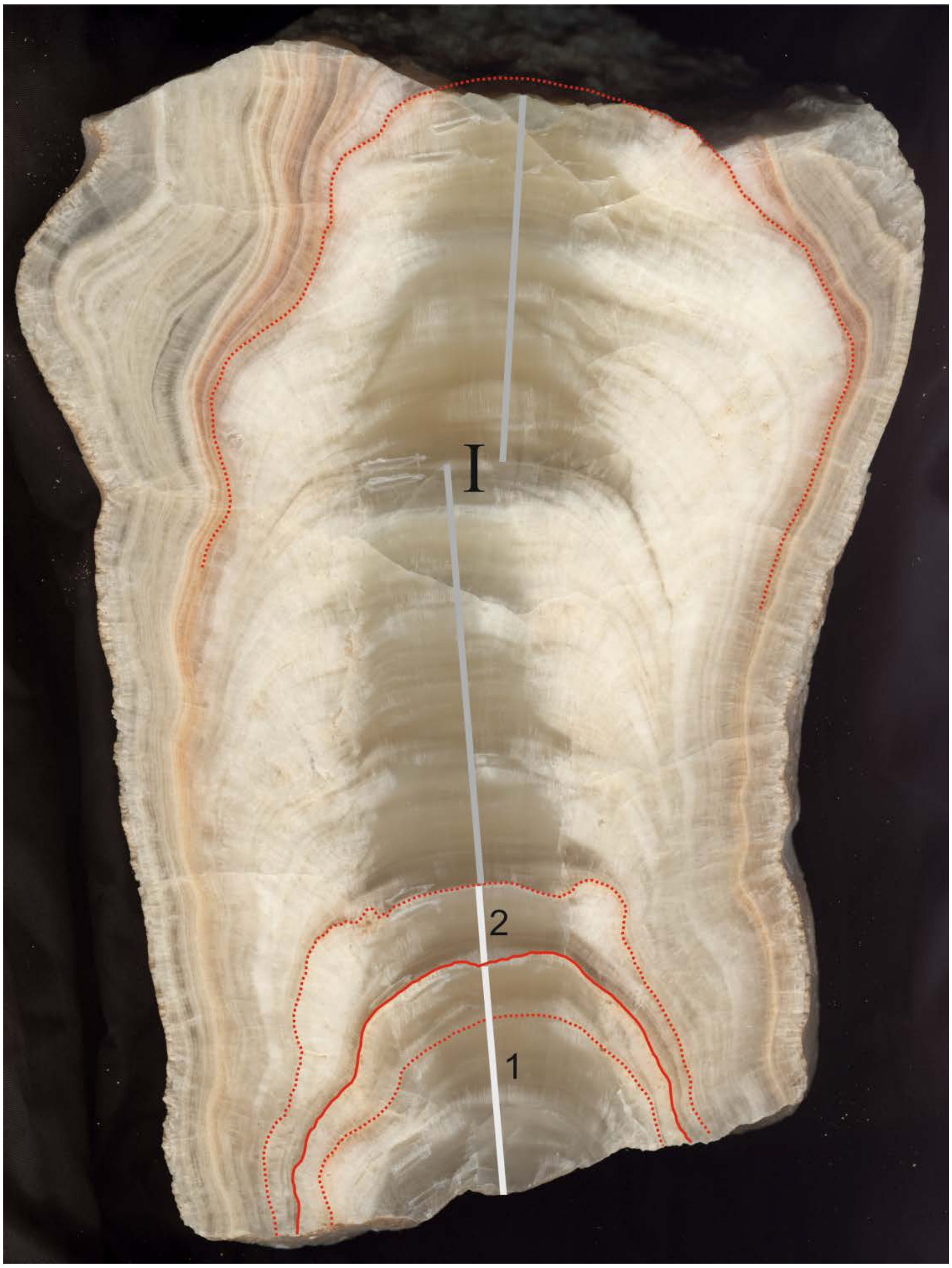

Fig. S3 a: SC-06-01 1, bottom piece.

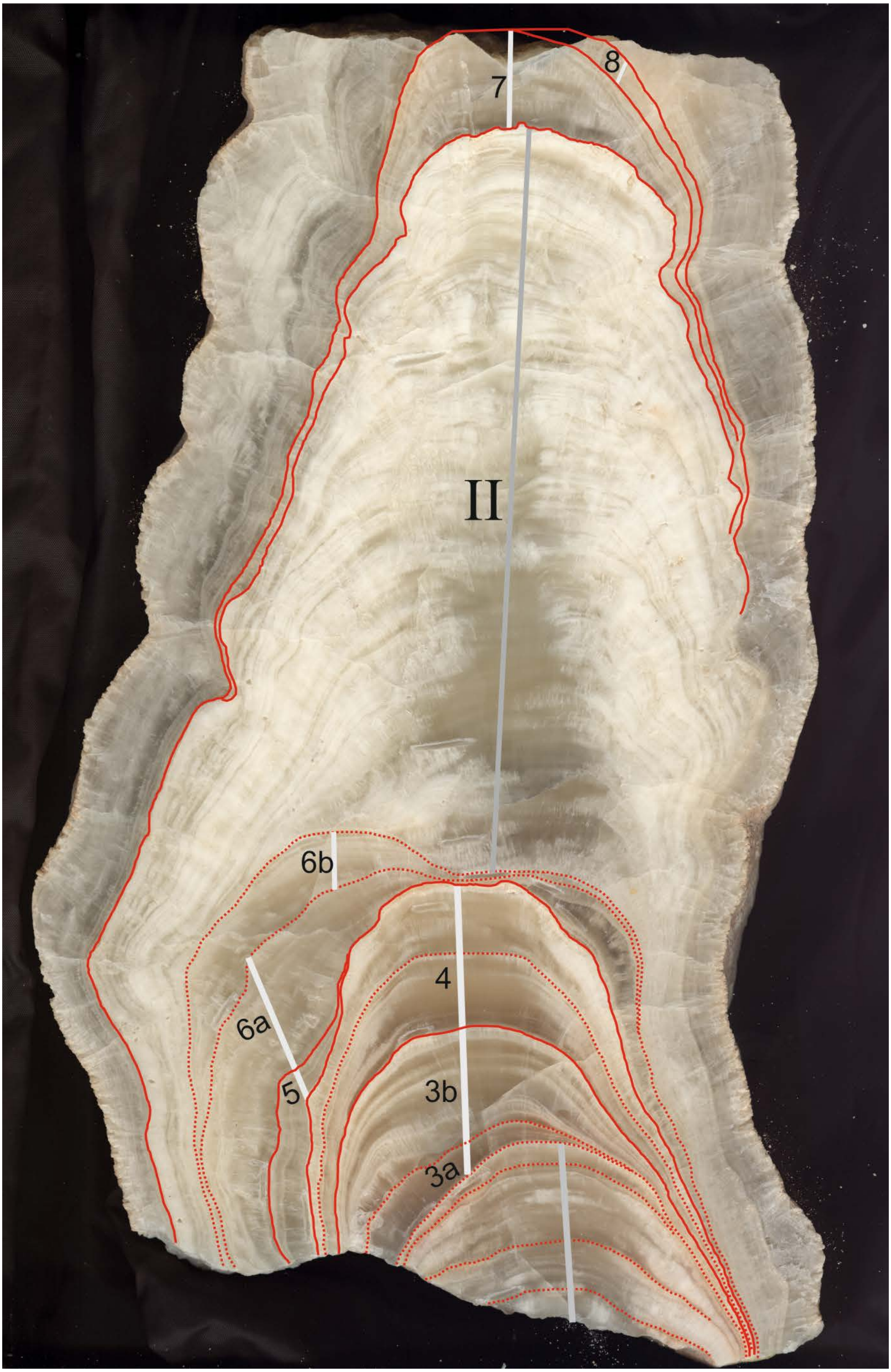

Fig. S3 b: SC-06-01 2, middle piece.

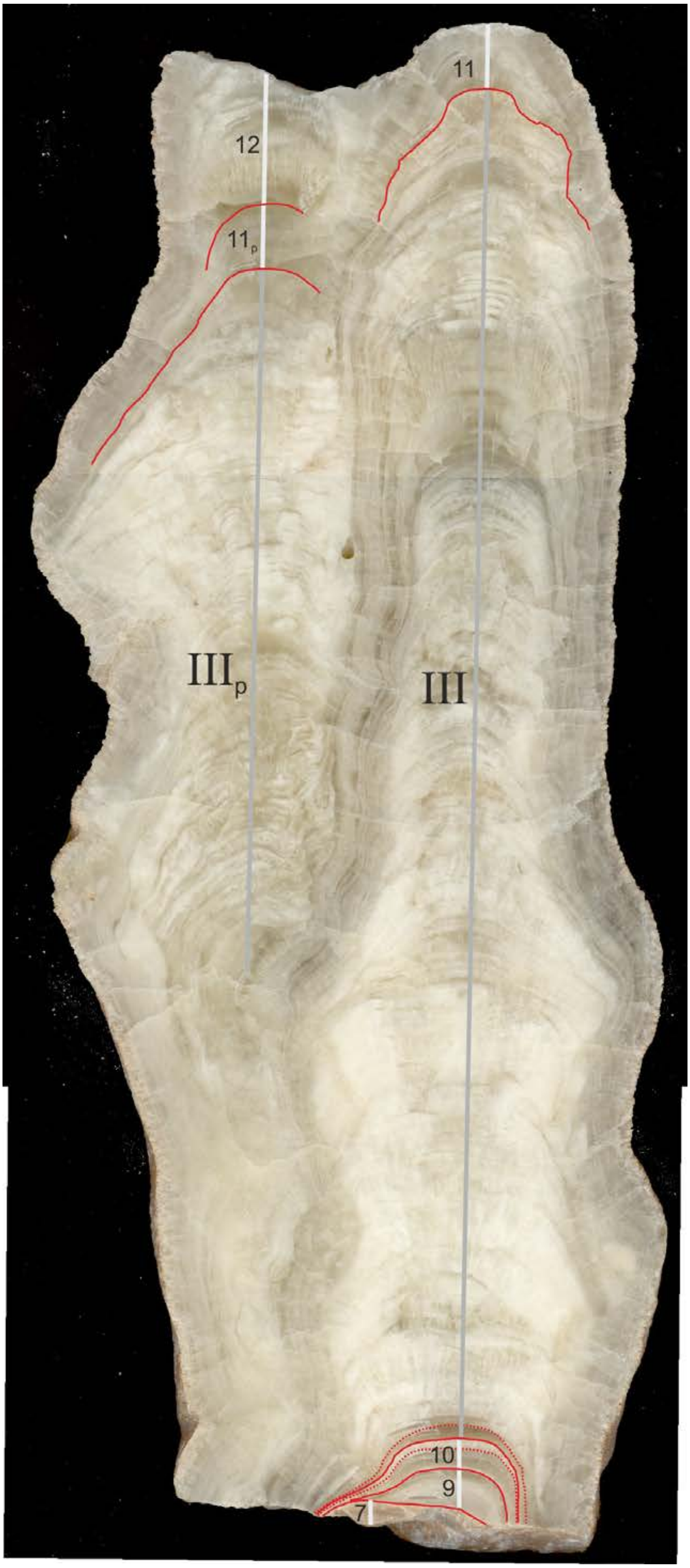

Fig. S3 c: SC-06-01 3, top piece.

U-series dating and age model

Stalagmite SC-06-01 consists of three broken pieces, which were cut along the growth axis and polished. Some sections were further cut into slabs along the growth axis for subsampling by micromill or with a wire saw. Sub samples for U-Th dating were either drilled using a hand held microdrill (powders) or small pieces were cut from slabs using a diamond wire saw (pieces). Sample masses for U-Th analyses varied between 10 and 180 mg.

Separation and purification of U and Th isotopes from the matrix was done using modified methods similar to Hoffmann [3]. Anion exchange chemistry consists of a double resin procedure with AG 1x8 used to separate U and Th followed by a first Th fraction purification using AG 1x8. Final purification of U and Th fractions is done using UTEVA resin. U and Th isotope compositions are measured by MC-ICPMS following procedures outlined in Hoffmann, et al. (2007) [4]. The following decay constants are used to calculate activity ratios: $\lambda_{238} = (1.55125 \pm 0.0017) \cdot 10^{-10}\ a^{-1}$ [5], $\lambda_{234} = (2.826 \pm 0.0056) \cdot 10^{-6}$ [6], $\lambda_{232} = (4.95 \pm 0.035) \cdot 10^{-11}$ [7], $\lambda_{230} = (9.1577 \pm 0.028) \cdot 10^{-6}$ [6].

The concentration of ^{238}U varies between 134 and 756 ng/g, with an average of 388 ng/g. U concentrations are found to be slightly higher in the bottom 300 mm and lower in the top part of the stalagmite. Initial $^{234}U/^{238}U$ activity ratios are quite consistent around 1.28. The calcite is clean and almost free of detrital components. All $^{230}Th/^{232}Th$ activity ratios are greater than 240 and the mean ratio is 6200, thus, a correction for detrital Th has no significant effect on the measured activity ratios and calculated ages (Table S1). However, a correction is usually applied where ^{232}Th is measurable, and a bulk earth value of the upper crust ($^{238}U/^{232}Th_{act} = 0.8 \pm 0.4$) [8] is used for detrital correction.

The stalagmite started forming around 66.5 ka and grew episodically until about 30.8 ka. The detailed chronology for SC-06-01 is based on 116 dating results along the growth axis (104 on the main axis and 12 on the twin axis at the top) and typical age uncertainties are in the range of 0.7 %. All uncertainties are at the 95 % confidence interval. Table S1 shows all analytical results and calculated ages. Fig. S4 shows all dating results versus distance on the growth axis, and Figures S5 a-c show details of the sections. The positions of red layers in the stalagmite are indicated by red lines. Dotted red lines represent red layers where no significant hiatus is obvious in the dating results.

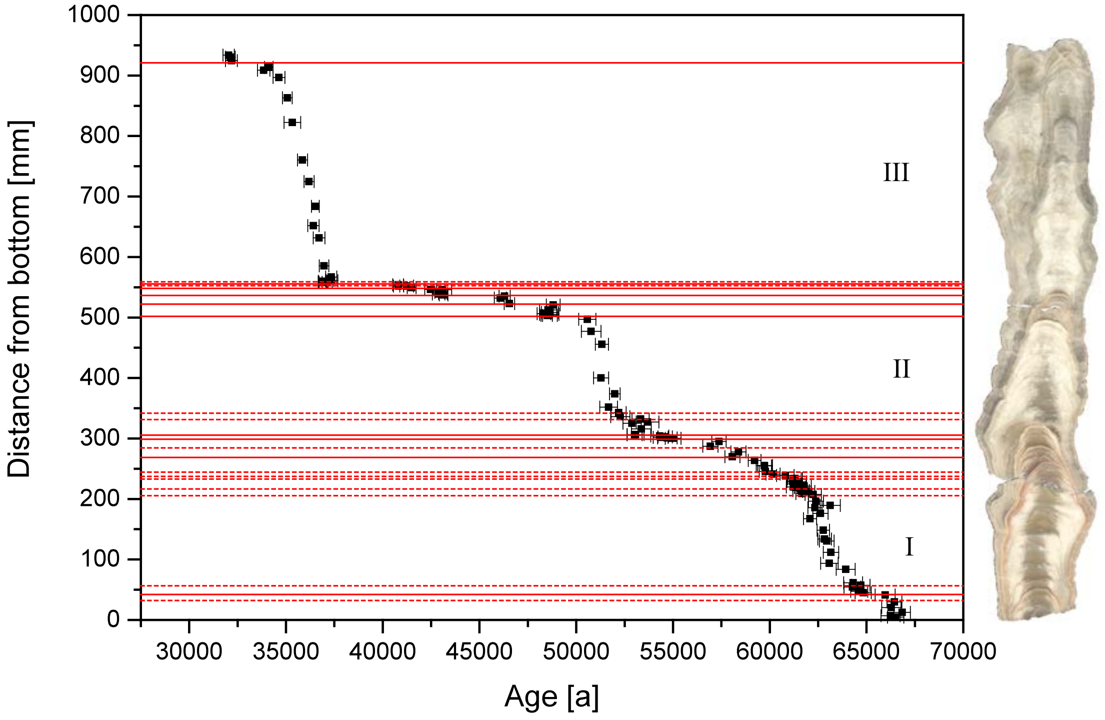

Fig. S4: Same as Fig. 2 in the main article. U-series dating results of SC-06-01, on the right cross section of stalagmite SC-06-01 scaled to the distances from bottom of the dating results. Dating results for the parallel section at the top of the stalagmite are not shown, see Fig. S6.

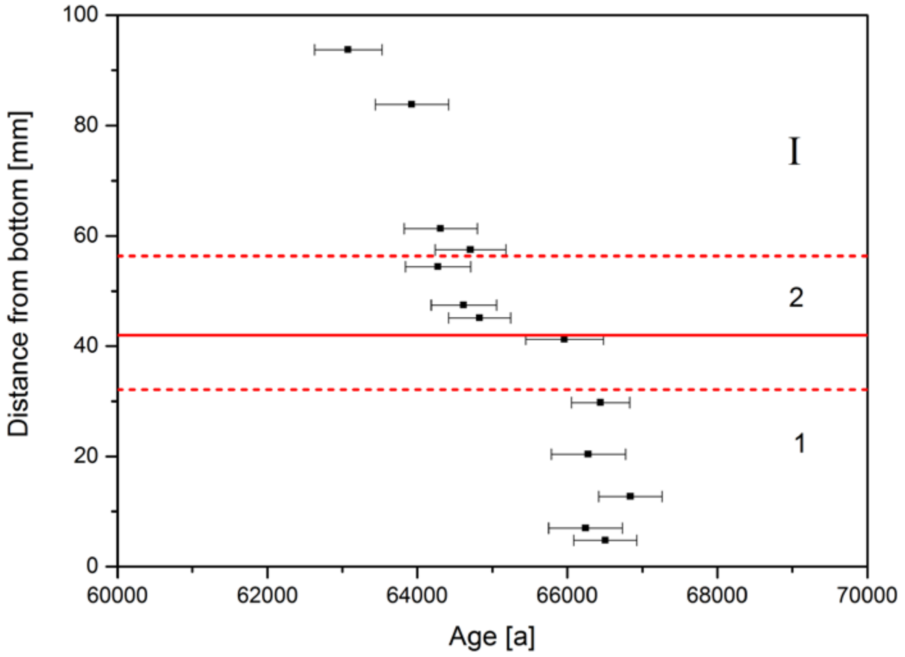

Fig. S5 a: Detail of Fig. S4, showing the dating results of sections with red layers.

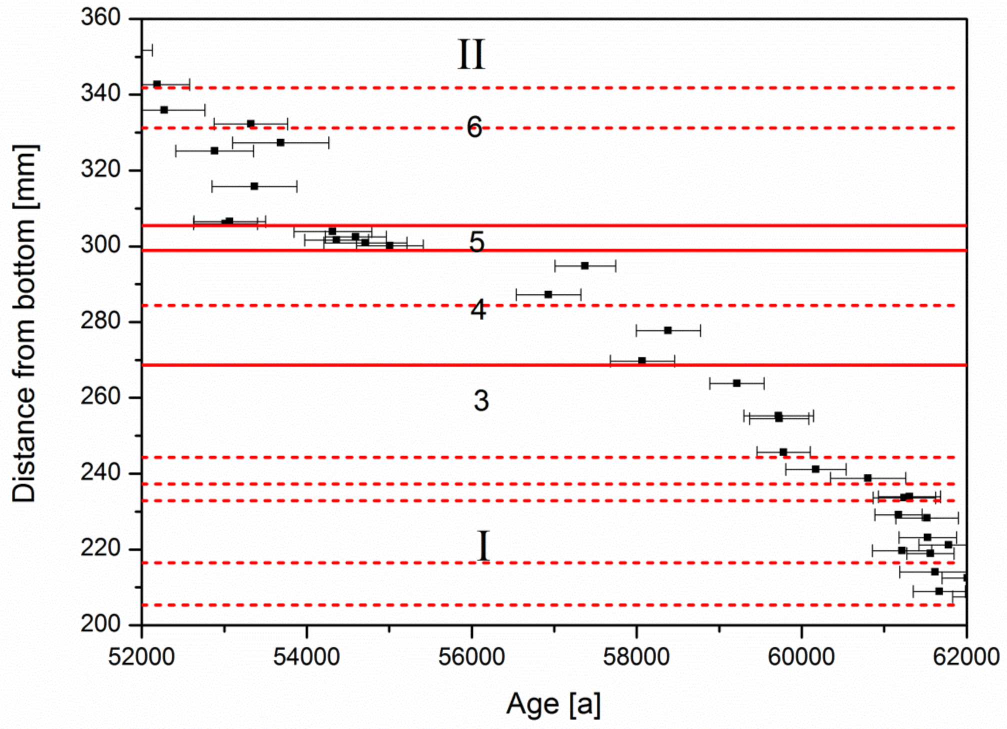

Fig. S5 b: Detail of Fig. S4, showing the dating results of sections with red layers.

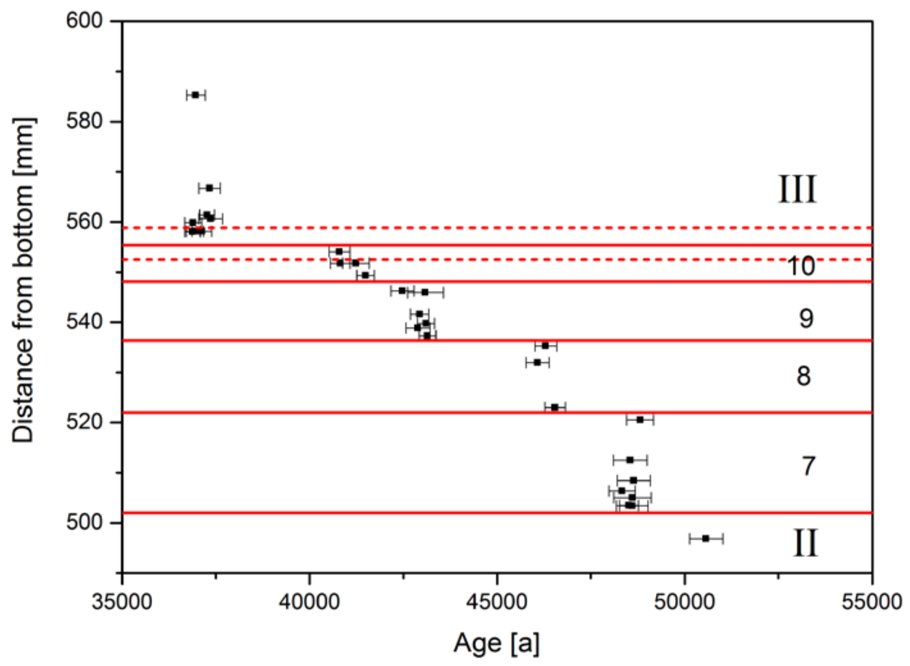

Fig. S5 c: Detail of Fig. S4, showing the dating results of sections with red layers.

Dating results of the parallel growth section at top of stalagmite are presented in Fig. S6. Here distances are shown from the top of the section. One dating result obtained on a sample taken from a boundary area between two sections including a red layer (between III_p and 11_p) is not in stratigraphic order.

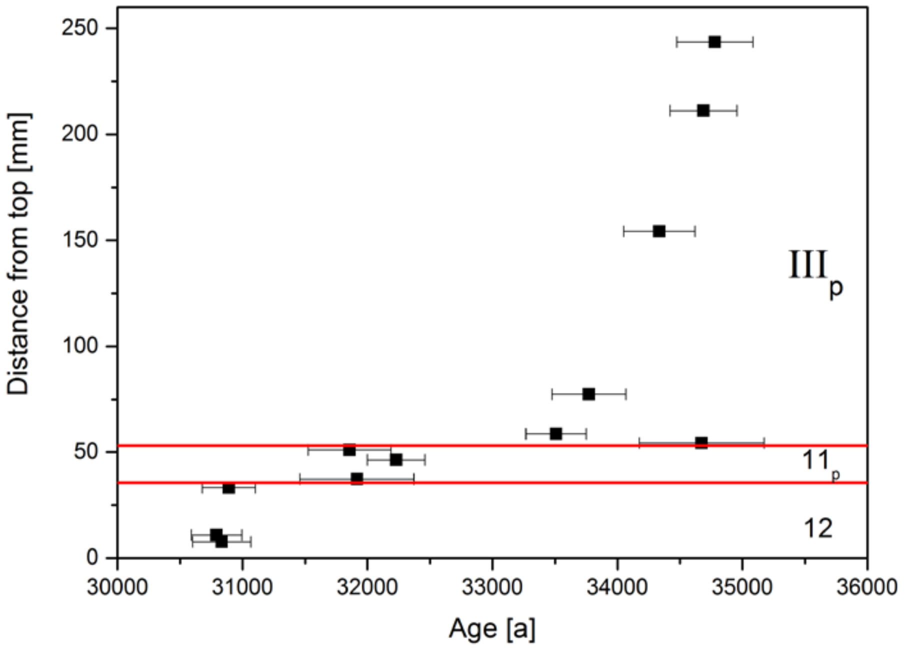

Fig. S6: U-Th dating results of the parallel growth axis at the top of SC-06-01.

Distance - age model

A distance-age model was generated for SC-06-01 using StalAge [9]. Distance age modeling was done sectionwise. The start and end of the sections / growth episodes are defined by the red layers associated with a detectable hiatus in the dating results. The distance-age model is shown in Fig. S7 a (main growth axis) and Fig. S7b (parallel growth axis at the top). The red lines indicate upper and lower 95% confidence levels of the age model.

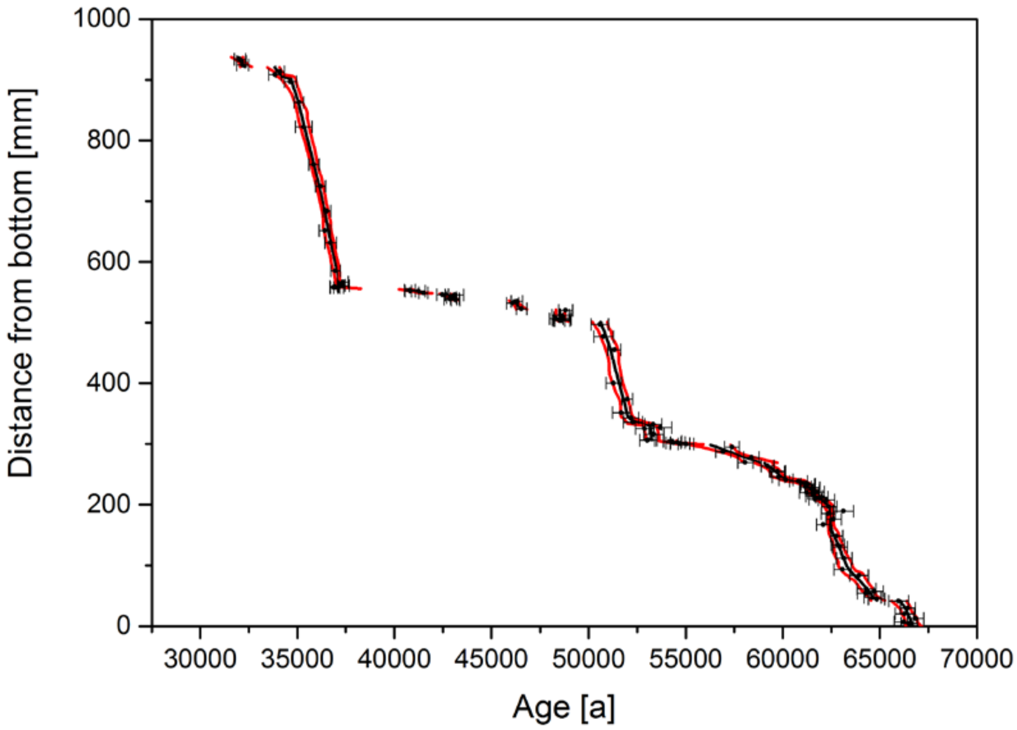

Fig. S7 a: Distance-age model for SC-06-01, main growth axis, derived using StalAge [9].

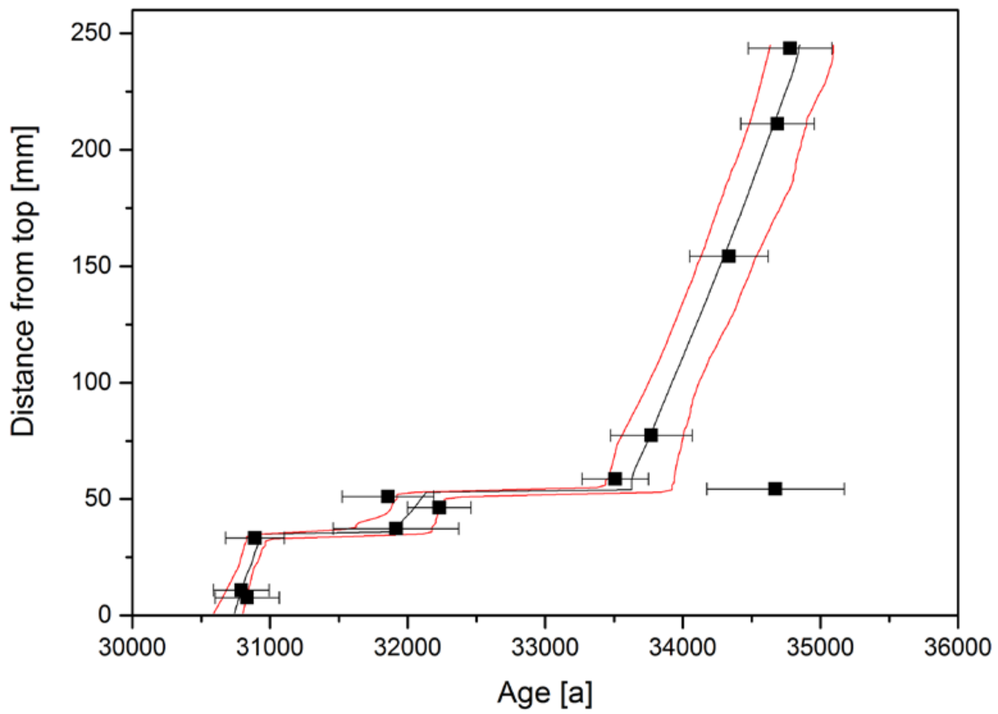

Fig. S7 b: Distance-age model for SC-06-01, parallel growth axis, derived using StalAge [9].

Stable isotopes results

Fig. S8 shows $\delta^{18}O$ and $\delta^{13}C$ results for the main axis of SC-06-01. Figures 8 and 9 a-d compare the Susah Cave oxygen isotope record ($\delta^{18}O_{SC}$) to the Greenland ice core oxygen isotope $\delta^{18}O_{NGRIP}$ [10]. The chronology for NGRIP older than 60 ka is based on GICC05modelext [11].

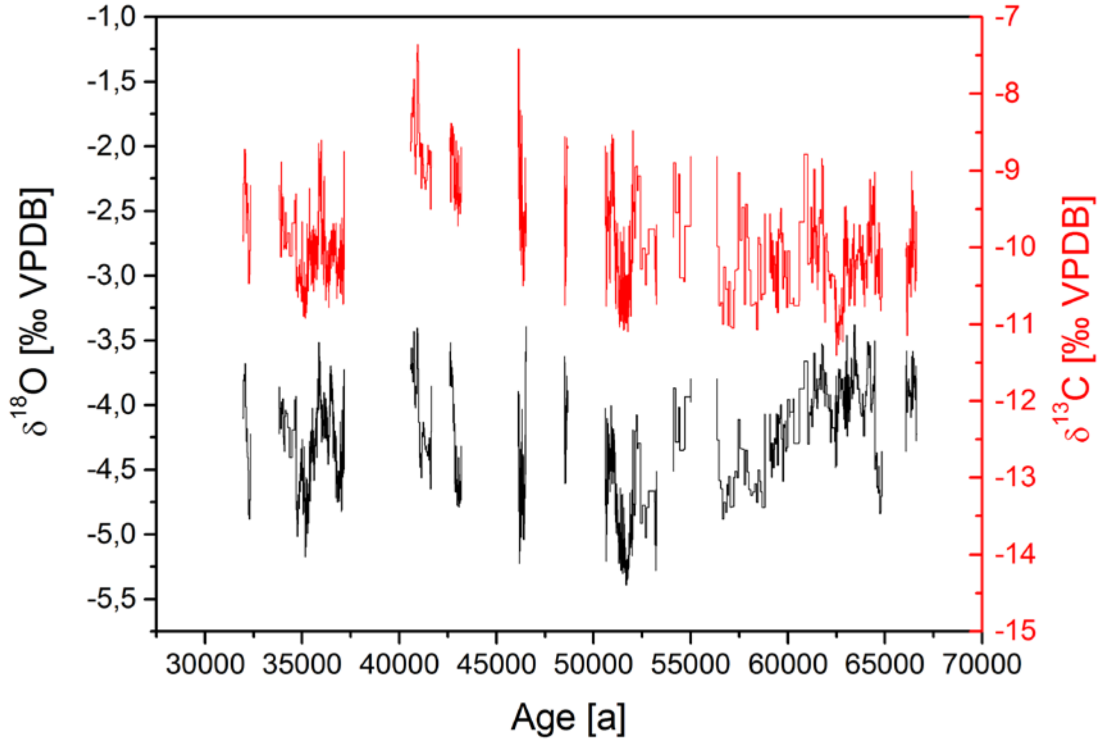

Fig. S8: Stable oxygen ($\delta^{18}O$) and carbon ($\delta^{13}C$) isotope results of stalagmite SC-06-01.

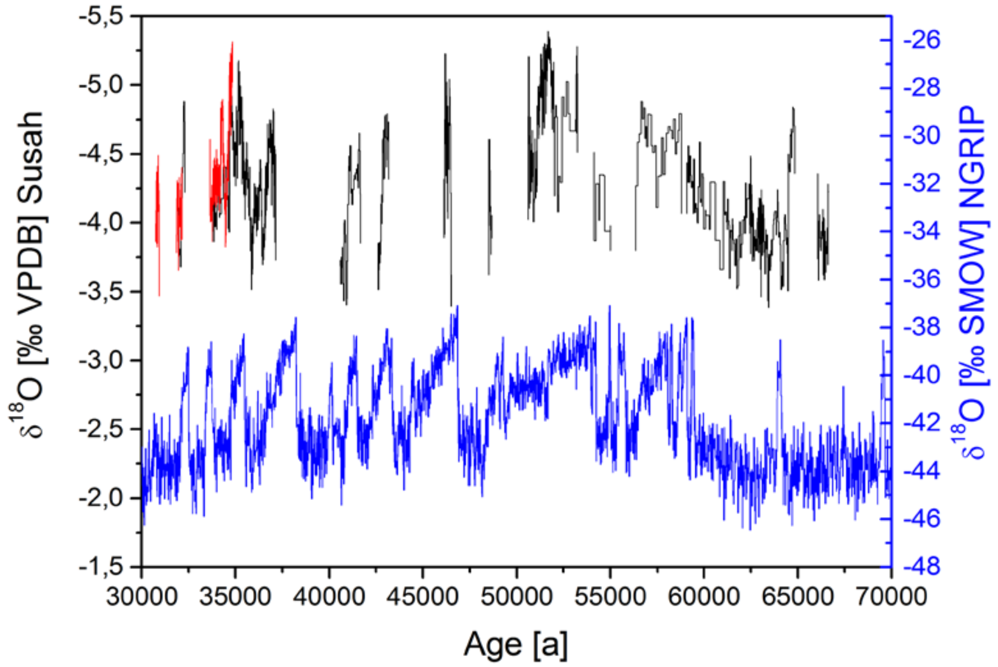

Fig. S9: Stable oxygen ($\delta^{18}O$) results of stalagmite SC-06-01 and Greenland ice core (NGRIP) [10,11] between 70 and 30 ka. The red line shows results on the parallel top section of SC-06-01.

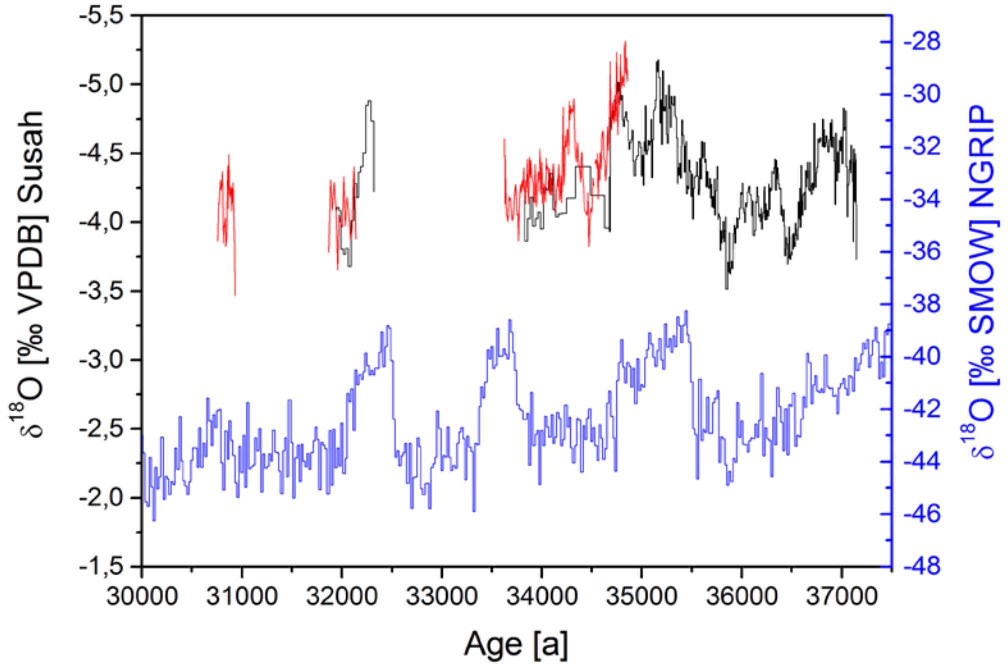

Fig. S10 a: Detail of Fig. S9, stable oxygen ($\delta^{18}O$) results of stalagmite SC-06-01 and Greenland ice core (NGRIP) [10,11] between 37.5 and 30 ka.

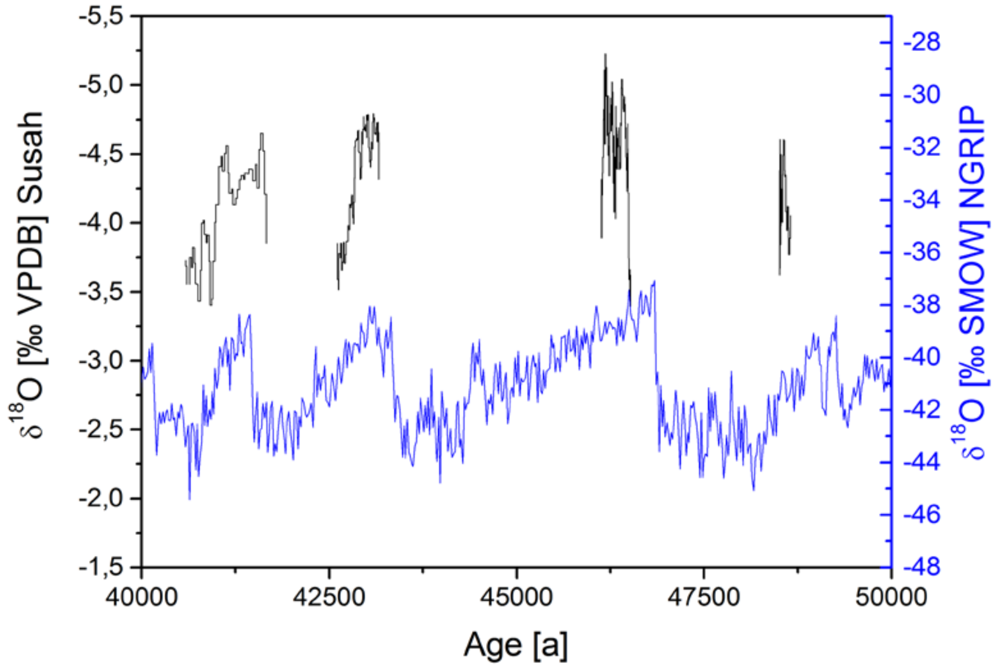

Fig. S10 b: Detail of Fig. S9, stable oxygen ($\delta^{18}O$) results of stalagmite SC-06-01 and Greenland ice core (NGRIP) [10,11] between 50 and 40 ka.

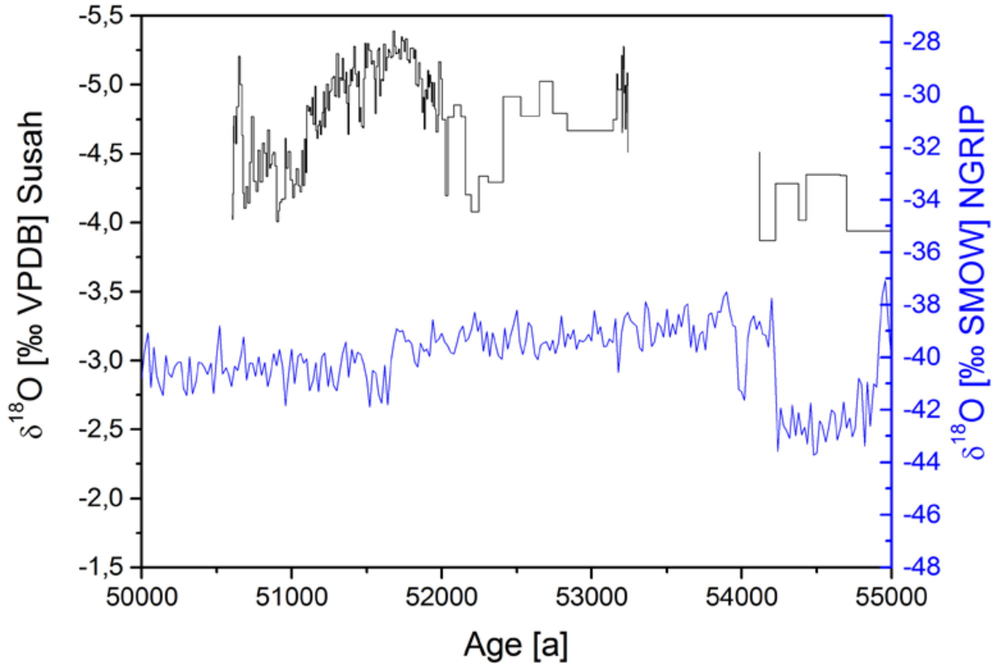

Fig. S10 c: Detail of Fig. S9, stable oxygen ($\delta^{18}O$) results of stalagmite SC-06-01 and Greenland ice core (NGRIP) [10,11] between 55 and 50 ka.

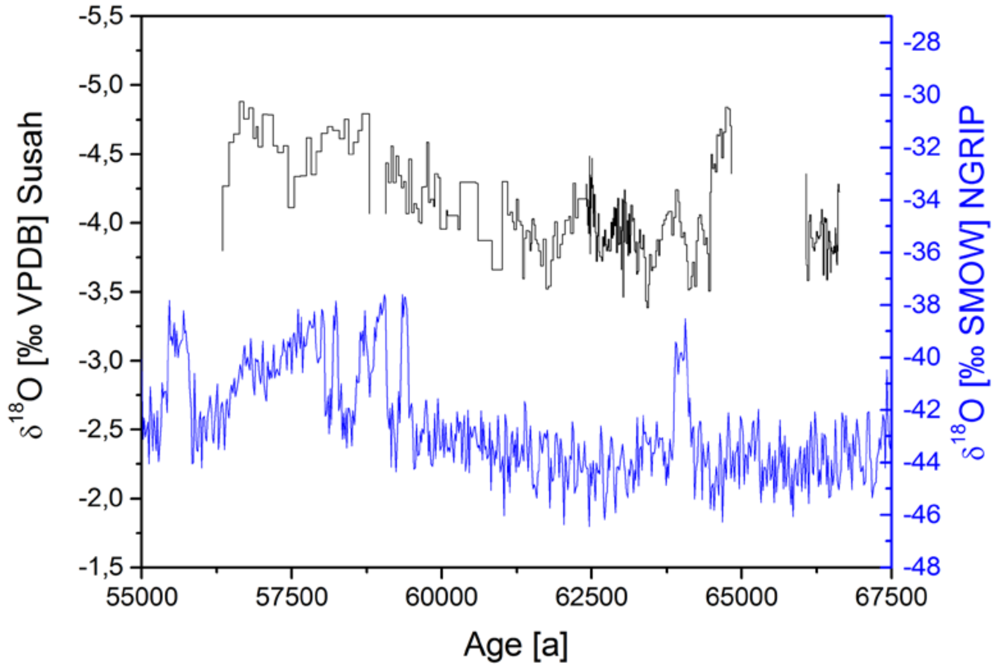

Fig. S10 d: Detail of Fig. S9, stable oxygen ($\delta^{18}O$) results of stalagmite SC-06-01 and Greenland ice core (NGRIP) [10,11] between 67.5 and 55 ka.

Comparison of timing of wet phases to Northern Hemisphere insolation changes

Growth phases of SC-06-01 are compared to insolation at 30°N in June and December as well as to the insolation gradient between 60°N and 30°N (the difference is calculated as insolation at 60°N minus insolation at 30°N). Insolation values are taken from Berger and Loutre (1991) [12]. Fig. S11 a shows summer insolation (June) at 30°N and Fig. S12 a winter insolation (December) at 30°N, both between 70 and 30 ka. SC-06-01 growth phases are indicated by the $\delta^{18}O$ record, additionally, the grey bars highlight the three main growth phases. Figures S11 b and S12 b show the 60°N-30°N insolation gradient between 70 and 30 ka.

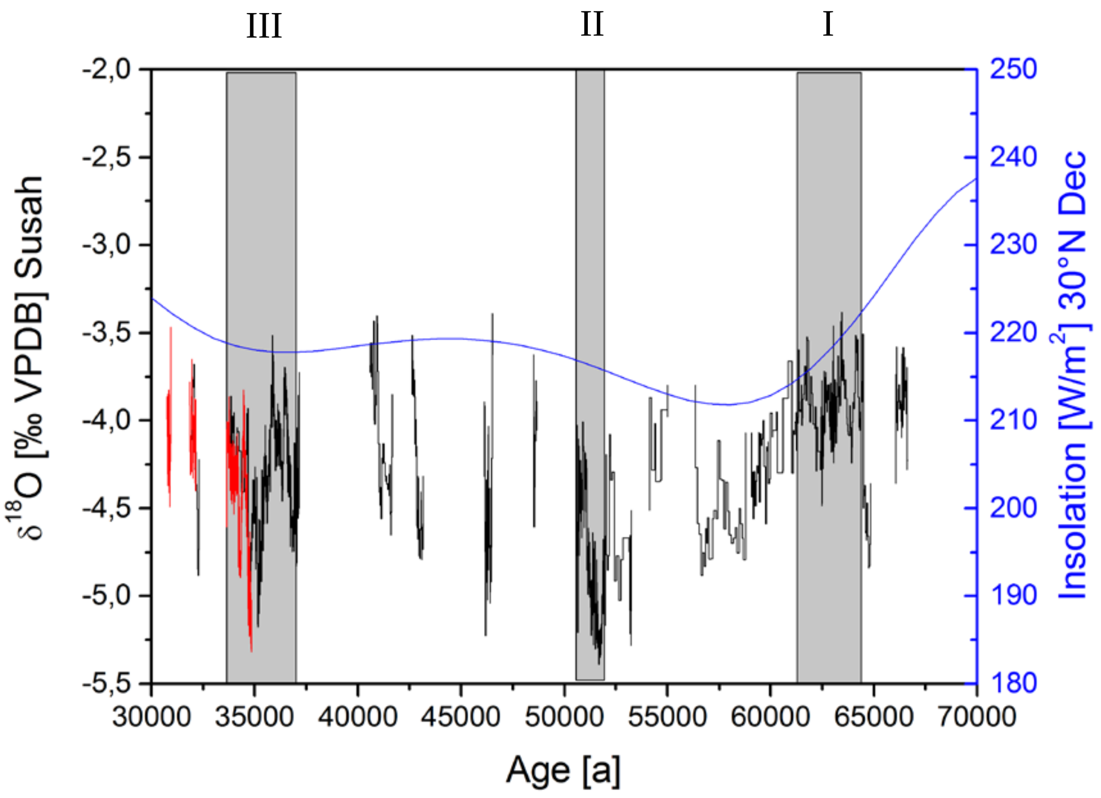

Fig. S11 a: SC-06-01 $\delta^{18}O$ record and Northern Hemisphere winter insolation at 30°N [12]. Grey bars indicate the three main growth phases (I, II and III) of SC-06-01.

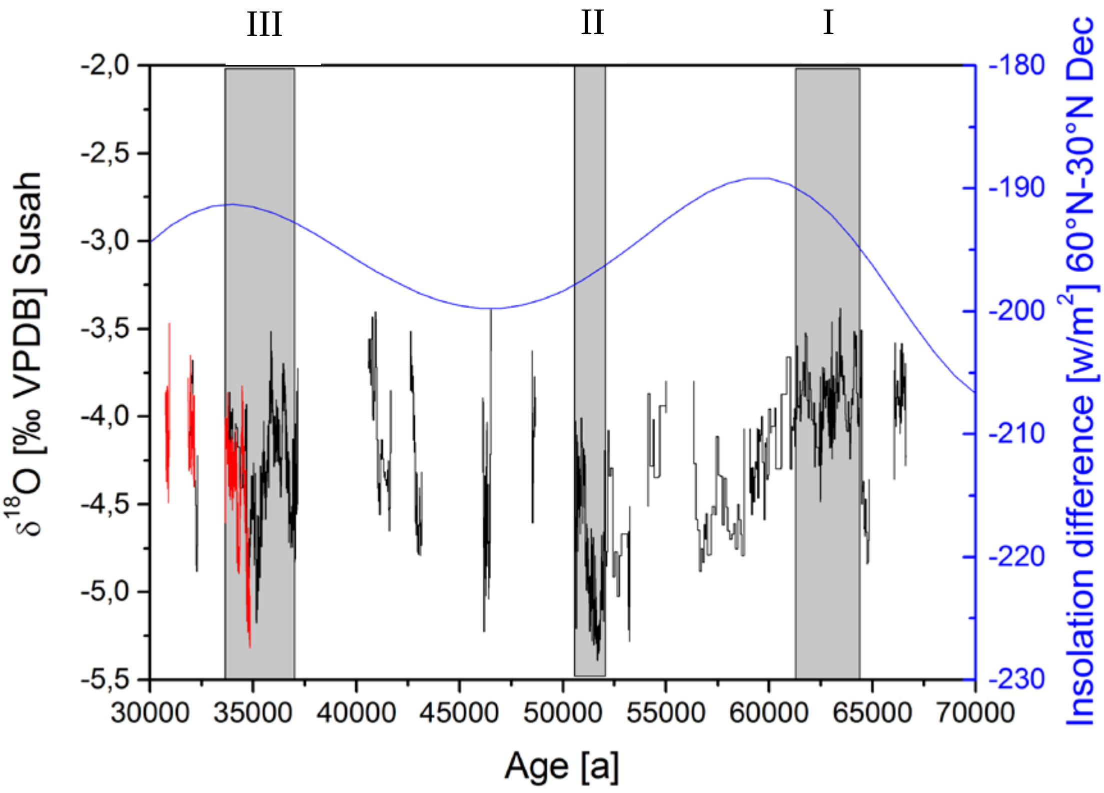

Fig. S11 b: SC-06-01 $\delta^{18}O$ record and difference of winter insolation between 30°N and 60°N (calculated as insolation at 60°N minus insolation at 30°N) [12]. Grey bars indicate the three main growth phases (I, II and III) of SC-06-01. Precession minimum results in two peaks of maximum December insolation gradient at 60°N relative to 30°N around 60 ka and 34 ka.

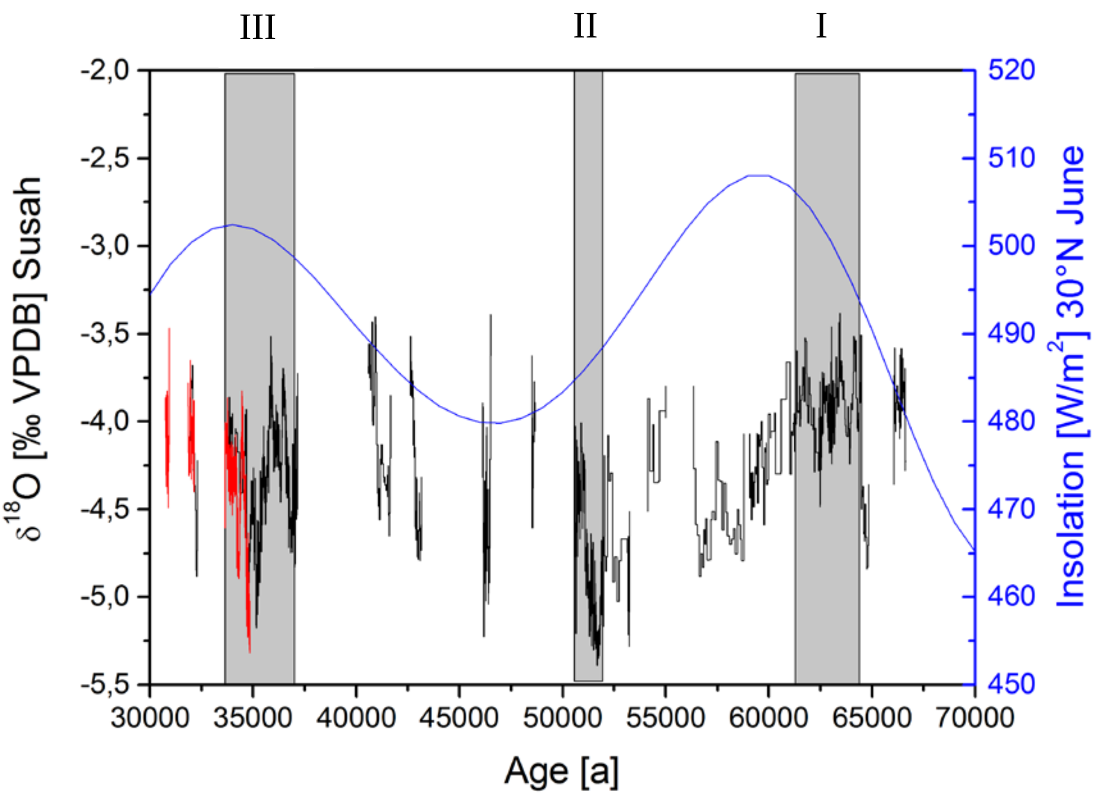

Fig. S12 a: SC-06-01 δ^{18}O record and Northern Hemisphere summer insolation at 30°N [12]. Grey bars indicate the three main growth phases (I, II and III) of SC-06-01.

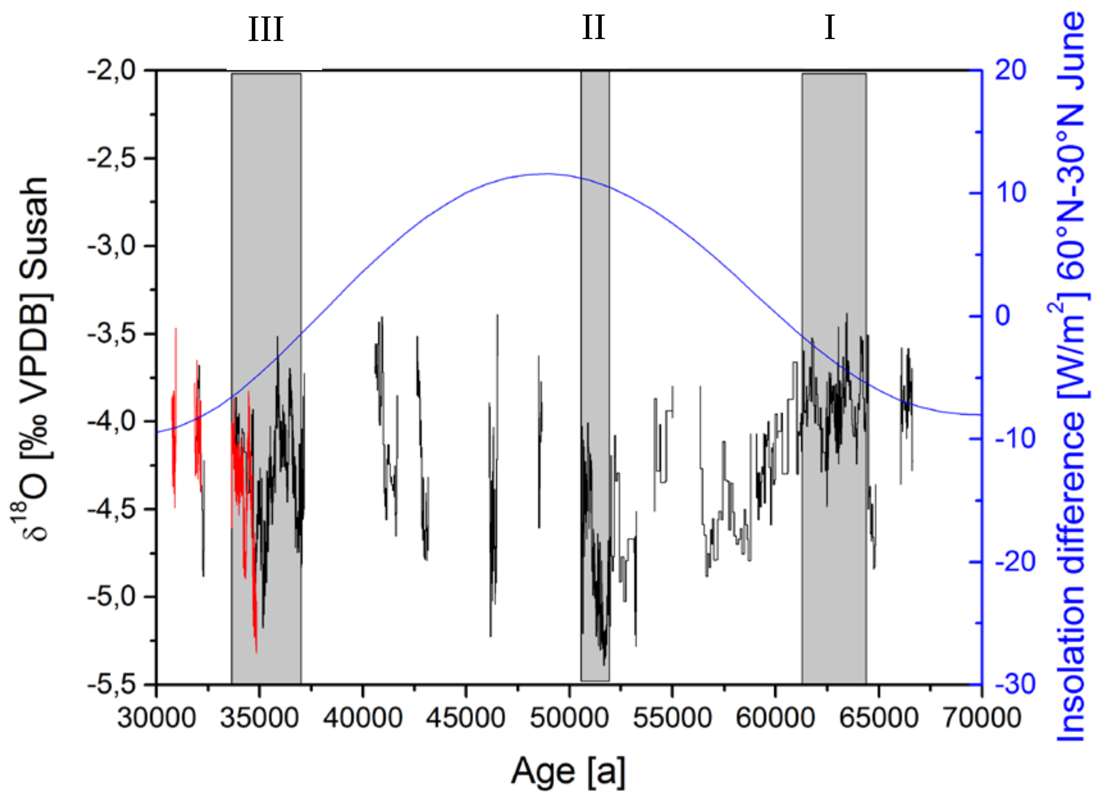

Fig S12 b: SC-06-01 $\delta^{18}O$ record and difference of summer insolation between 30°N and 60°N (calculated as insolation at 60°N minus insolation at 30°N) [12]. Grey bars indicate the three main growth phases (I, II and III) of SC-06-01. Obliquity maximum results in a maximum June insolation gradient at 60°N relative to 30°N around 50 ka.

Hendy test results

Results of 18 Hendy tests (H1 - H18) are shown in the figures below. H1 to H15 are done on the central axis, H16 - H18 on the parallel axis of the top piece.

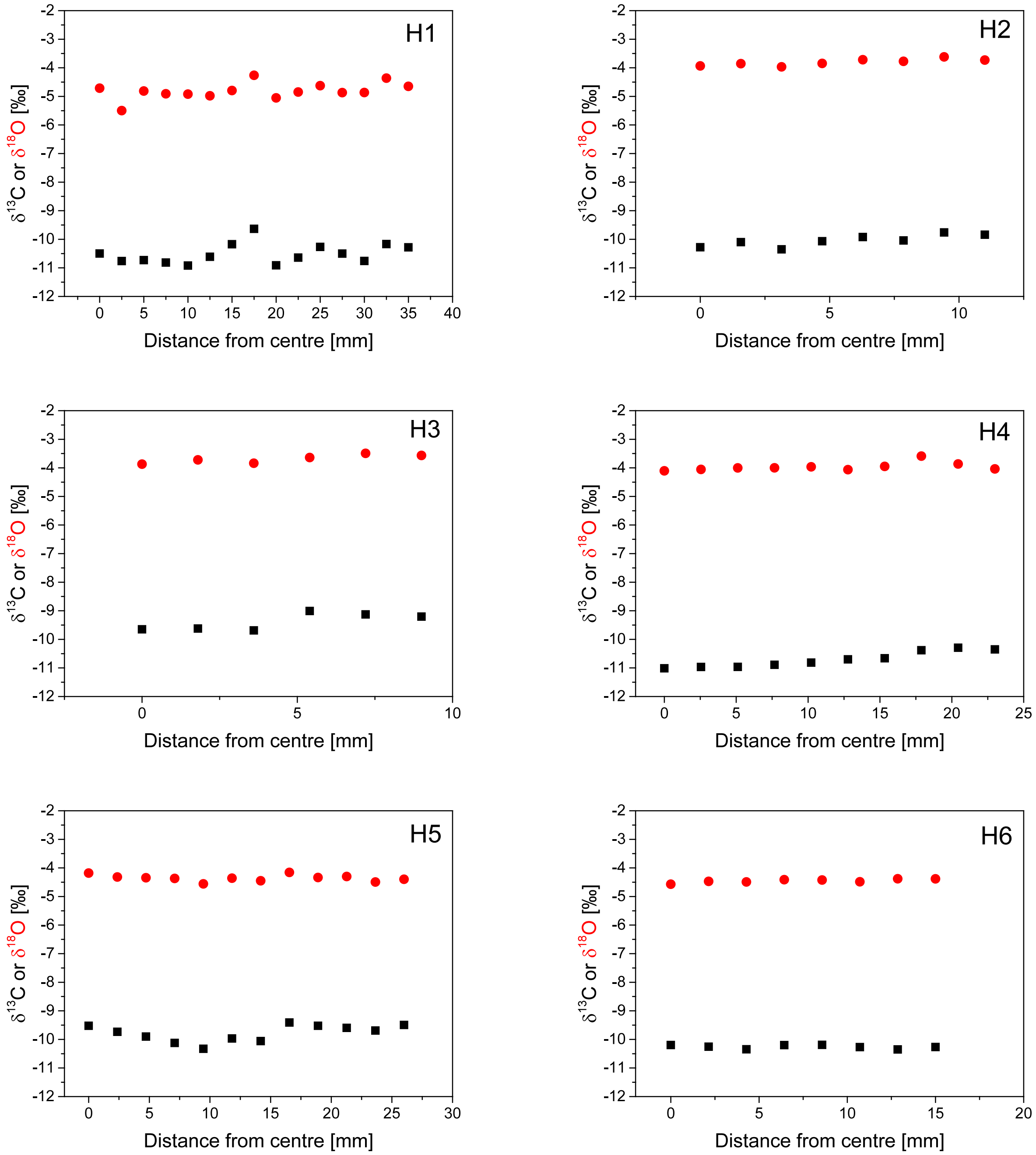

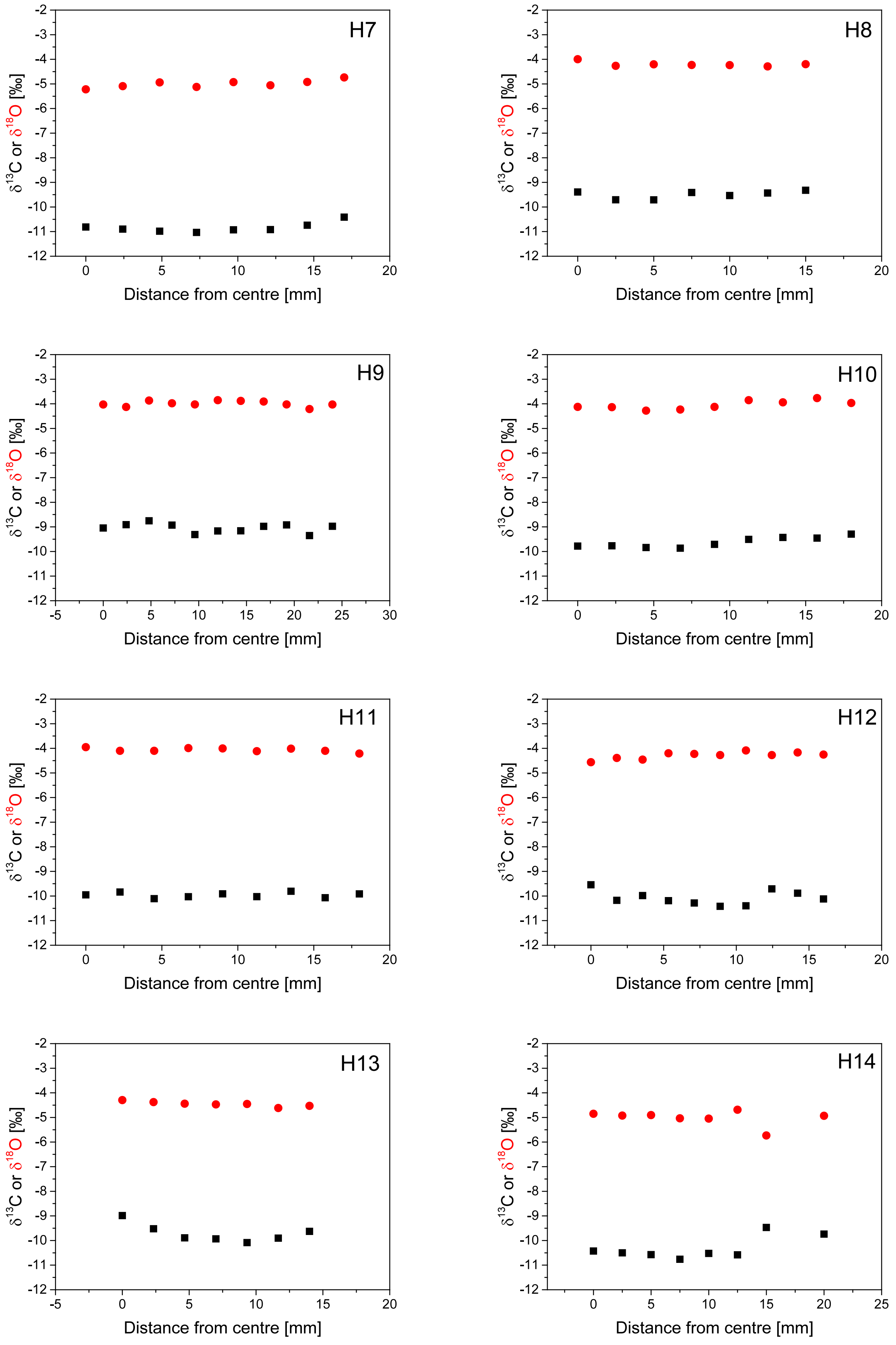
H7
H8
H9
H10
H11
H12
H13
H14
δ13C or δ18O [‰]
Distance from centre [mm]

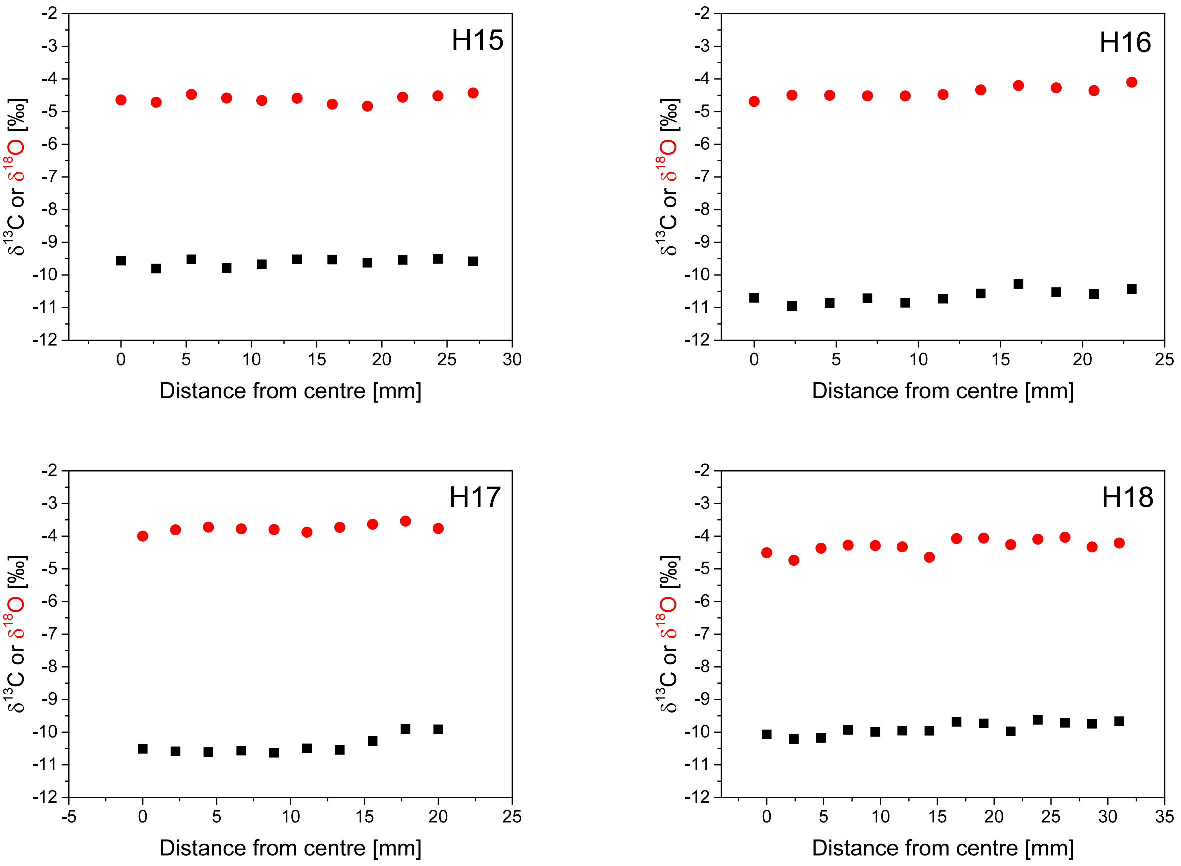
H15
H16
H17
H18
δ13C or δ18O [‰]
Distance from centre [mm]

Table S1. U-series results.

spl ID	***distance from base***	^{238}U	±	^{232}Th	±	$^{230}Th/^{232}Th$	±	$^{230}Th/^{238}U$	±	$^{234}U/^{238}U$	±	***age***	±	$^{234}U/^{238}U_{ini}$	±
	[mm]	(ng/g)		(ng/g)		activity ratio		activity ratio		activity ratio		(ka)		activity ratio	
SC-06-01 1 #03	4.8 (b)	405.9	3.4	0.285	0.003	2520	17	0.5798	0.0023	1.2486	0.0029	66.51	0.42	1.3000	0.0033
SC-06-01 1 #13	7.0	455.0	3.8	0.122	0.002	6639	63	0.5828	0.0030	1.2582	0.0025	66.24	0.49	1.3114	0.0029
SC-06-01 1 #06	12.7	445.1	3.5	0.110	0.002	7230	61	0.5844	0.0025	1.2537	0.0025	66.84	0.42	1.3065	0.0028
SC-06-01 1 #12	20.4	358.3	3.1	0.083	0.002	7646	102	0.5827	0.0029	1.2575	0.0028	66.28	0.50	1.3106	0.0032
SC-06-01 1 #21	29.8	524.4	5.5	0.176	0.003	5315	42	0.5827	0.0022	1.2554	0.0023	66.44	0.39	1.3082	0.0027
SC-06-01 1 #08	41.2	614.4	4.9	0.163	0.002	6672	51	0.5783	0.0029	1.2529	0.0036	65.96	0.52	1.3048	0.0041
SC-06-01 1 #04	45.1	464.0	3.5	0.156	0.002	5138	41	0.5665	0.0024	1.2439	0.0025	64.83	0.41	1.2929	0.0029
SC-06-01 1 #22	47.5	473.9	3.6	0.227	0.011	3643	90	0.5704	0.0027	1.2547	0.0020	64.62	0.44	1.3058	0.0024
SC-06-01 1 #07	54.4	474.5	3.3	0.215	0.002	3811	27	0.5645	0.0025	1.2471	0.0029	64.27	0.43	1.2964	0.0033
SC-06-01 1 #05	57.5	375.5	3.1	0.487	0.006	1331	10	0.5646	0.0029	1.2413	0.0024	64.71	0.47	1.2899	0.0028
SC-06-01 1 #14	61.3	439.4	3.5	0.092	0.002	8265	97	0.5661	0.0030	1.2501	0.0026	64.31	0.49	1.3000	0.0029
SC-06-01 1 #15	83.8	507.9	3.7	0.054	0.002	16032	276	0.5588	0.0030	1.2402	0.0025	63.93	0.49	1.2877	0.0029
SC-06-01 1 #23	93.8	510.4	4.5	0.131	0.010	6567	140	0.5534	0.0028	1.2405	0.0021	63.08	0.45	1.2875	0.0024
SC-06-01 1 #19	111.9	520.4	3.9	0.147	0.003	5980	59	0.5516	0.0024	1.2356	0.0020	63.16	0.40	1.2817	0.0023
SC-06-01 1 #09	130.6	510.8	3.2	0.120	0.001	7172	55	0.5508	0.0022	1.2370	0.0024	62.94	0.38	1.2832	0.0027
SC-06-01 1 #10	133.8	519.6	3.0	0.025	0.001	34992	444	0.5499	0.0020	1.2367	0.0024	62.83	0.35	1.2827	0.0028
SC-06-01 1 #20	148.4	673.7	5.5	0.036	0.002	31048	398	0.5487	0.0019	1.2352	0.0022	62.76	0.32	1.2809	0.0025

SC-06-01 1 #24	167.5	506.6	4.2	0.187	0.004	4470	48	0.5395	0.0020	1.2251	0.0022	62.08	0.34	1.2683	0.0025
SC-06-01 1 #16	176.2	549.9	4.2	0.036	0.002	25529	658	0.5457	0.0023	1.2309	0.0025	62.62	0.39	1.2756	0.0029
SC-06-01 1 #25	185.6	697.1	4.8	0.206	0.003	5599	49	0.5423	0.0022	1.2274	0.0019	62.34	0.36	1.2713	0.0022
SC-06-01 1 #02	189.5	583.7	5.3	0.115	0.002	8377	114	0.5410	0.0031	1.2140	0.0025	63.12	0.52	1.2558	0.0029
SC-06-01 1 #11	196.5	556.7	4.6	0.043	0.001	21342	244	0.5434	0.0023	1.2293	0.0025	62.39	0.39	1.2735	0.0029
SC-06-01 2 #06	207.5	351.3	2.8	1.107	0.012	527	3	0.5433	0.0025	1.2300	0.0025	62.25	0.42	1.2745	0.0029
SC-06-01 2 #25	208.9	482.3	1.6	0.049	0.001	15996	150	0.5340	0.0019	1.2192	0.0020	61.67	0.32	1.2610	0.0023
SC-06-01 2 #26	212.4	301.2	1.2	0.046	0.001	10794	116	0.5392	0.0018	1.2255	0.0020	62.01	0.31	1.2687	0.0023
SC-06-01 2 #18	214.1	294.1	2.5	0.151	0.004	3209	43	0.5400	0.0026	1.2325	0.0024	61.62	0.43	1.2768	0.0027
SC-06-01 2 #27	218.9	587.1	2.3	0.022	0.001	43570	520	0.5375	0.0017	1.2283	0.0016	61.56	0.29	1.2716	0.0019
SC-06-01 2 #19	219.7	526.7	4.7	0.126	0.004	6829	102	0.5360	0.0022	1.2300	0.0022	61.22	0.36	1.2734	0.0025
SC-06-01 2 #28	221.2	605.4	24.5	0.151	0.008	6604	52	0.5398	0.0022	1.2299	0.0022	61.78	0.36	1.2737	0.0025
SC-06-01 2 #29	223.2	408.0	1.7	0.093	0.001	7189	57	0.5379	0.0022	1.2295	0.0018	61.53	0.35	1.2730	0.0021
SC-06-01 2 #20	228.3	532.6	5.6	0.136	0.007	6442	103	0.5385	0.0023	1.2309	0.0020	61.52	0.38	1.2747	0.0023
SC-06-01 2 #30	229.1	620.6	2.5	0.053	0.001	19204	191	0.5367	0.0017	1.2319	0.0019	61.17	0.29	1.2757	0.0021
SC-06-01 2 #31	233.6	475.3	2.6	0.076	0.001	10272	89	0.5376	0.0024	1.2329	0.0020	61.24	0.38	1.2769	0.0023
SC-06-01 2 #07	233.9	539.8	4.2	0.130	0.002	6864	55	0.5404	0.0023	1.2381	0.0023	61.31	0.38	1.2832	0.0027
SC-06-01 2 #01	238.8	549.2	4.4	0.349	0.004	2584	21	0.5371	0.0028	1.2381	0.0025	60.81	0.45	1.2828	0.0028
SC-06-01 2 #21	241.1	738.5	7.2	0.353	0.007	3422	31	0.5354	0.0023	1.2437	0.0019	60.17	0.37	1.2889	0.0022
SC-06-01 2 #22	245.7	736.6	6.6	0.642	0.009	1855	14	0.5290	0.0020	1.2354	0.0021	59.78	0.32	1.2788	0.0024
SC-06-01 2 #23	254.5	431.7	3.6	0.609	0.008	1155	9	0.5331	0.0023	1.2451	0.0020	59.73	0.36	1.2903	0.0023
SC-06-01 2 #32	255.3	388.1	1.9	0.137	0.002	4624	48	0.5323	0.0027	1.2438	0.0021	59.72	0.42	1.2887	0.0024

SC-06-01 2 #24	263.8	521.2	4.3	0.074	0.003	11347	143	0.5276	0.0021	1.2409	0.0020	59.22	0.33	1.2849	0.0023
SC-06-01 2 #08	269.7	460.8	4.1	0.058	0.001	12578	140	0.5192	0.0024	1.2398	0.0027	58.07	0.39	1.2826	0.0031
SC-06-01 2 #44	277.7	517.9	6.5	0.291	0.004	2838	22	0,5221	0,0024	1.2413	0.0027	58.38	0.39	1.2846	0.0030
SC-06-01 2 #45	287.2	636.2	10.4	0.443	0.008	2279	16	0,5198	0,0026	1.2591	0.0026	56.93	0.39	1.3044	0.0028
SC-06-01 2 #03	294.9	625.4	4.2	0.275	0.004	3587	35	0.5167	0.0023	1.2451	0.0027	57.38	0.37	1.2882	0.0030
SC-06-01 2 #11	300.2	378.7	3.6	0.126	0.002	4560	39	0.4967	0.0025	1.2372	0.0031	55.01	0.41	1.2771	0.0035
SC-06-01 2 #36	300.9	416.7	5.1	0.418	0.009	1506	16	0.4939	0.0033	1.2354	0.0031	54.71	0.50	1.2748	0.0035
SC-06-01 2 #48	301.7	477.5	6.0	0.481	0.008	1498	11	0.4934	0.0025	1.2401	0.0025	54.36	0.39	1.2800	0.0028
SC-06-01 2 #49	302.5	391.8	5.8	0.412	0.008	1443	14.	0.4965	0.0024	1.2433	0.0028	54.59	0.37	1.2840	0.0032
SC-06-01 2 #50	303.9	328.6	6.2	0.606	0,013	821	8	0.4954	0.0031	1.2452	0.0030	54.32	0.47	1.2860	0.0034
SC-06-01 2 #40	306.0	454.9	8.3	1.036	0.024	649	5	0.4840	0.0026	1.2401	0.0027	53.01	0.39	1.2791	0.0030
SC-06-01 2 #12	306.5	351.4	2.9	0.097	0.001	5388	59	0.4881	0.0027	1.2500	0.0038	53.07	0.43	1.2905	0.0042
SC-06-01 2 #37	315.8	348.3	3.7	0.198	0.007	2602	45	0.4841	0.0035	1.2348	0.0028	53.37	0.51	1.2731	0.0032
SC-06-01 2 #43	325.2	274.2	2.3	0.111	0.003	3632	40	0.4820	0.0032	1.2384	0.0027	52.88	0.47	1.2768	0.0030
SC-06-01 2 #38	327.3	279.2	3.1	0.956	0.015	437	5	0.4899	0.0038	1.2422	0.0041	53.66	0.58	1.2821	0.0046
SC-06-01 2 #13	332.3	349.4	2.8	0.153	0.002	3382	27	0.4858	0.0028	1.2399	0.0037	53.32	0.45	1.2789	0.0042
SC-06-01 2 #39	335.9	284.1	2.8	0.267	0.007	1571	23	0.4827	0.0034	1.2505	0.0031	52.27	0.49	1.2905	0.0035
SC-06-01 2 #14	342.7	276.8	2.4	0.062	0.001	6524	74	0.4808	0.0026	1.2477	0.0029	52.19	0.39	1.2871	0.0033
SC-06-01 2 #04	351.7	301.5	2.3	0.090	0.002	4893	49	0.4769	0.0031	1.2475	0.0031	51.67	0.46	1.2864	0.0035
SC-06-01 2 #16	373.6	328.5	1.7	0.032	0.001	14695	149	0.4737	0.0018	1.2340	0.0021	51.99	0.28	1.2710	0.0024
SC-06-01 2 #41	400.0	331.3	2.7	0.133	0.003	3585	38	0.4702	0.0027	1.2378	0.0022	51.28	0.39	1.2750	0.0025
SC-06-01 2 #17	455.6	233.7	1.3	0.069	0.001	4894	39	0.4737	0.0023	1.2458	0.0025	51.32	0.34	1.2842	0.0028

SC-06-01 2 #42	477.0	241.2	3.4	0.182	0.005	1923	22	0.4739	0.0036	1.2563	0.0030	50.77	0.51	1.2959	0.0033
SC-06-01 2 #05	496.8	295.4	2.4	0.095	0.002	4437	56	0.4664	0.0030	1.2414	0.0033	50.57	0.44	1.2785	0.0037
SC-06-01 2 #02	503.4	338.9	2.6	0.298	0.003	1579	16	0.4537	0.0030	1.2462	0.0027	48.60	0.42	1.2825	0.0031
SC-06-01 2 #33	503.5	387.3	15.4	0.036	0.002	14935	167	0.4538	0.0017	1.2487	0.0020	48.51	0.25	1.2852	0.0022
SC-06-01 2 #46	505.0	330.8	5.1	1.234	0.020	410	4	0.4555	0,0036	1.2497	0.0030	48.61	0.50	1.2867	0.0033
SC-06-01 2 #34	506.3	269.5	10.3	0.108	0.008	3468	56	0.4531	0.0025	1.2502	0.0025	48.33	0.35	1.2869	0.0028
SC-06-01 2 #35	508.5	290.8	11.9	0.139	0.010	2924	52	0.4557	0.0031	1.2509	0.0028	48.64	0.44	1.2880	0.0032
SC-06-01 2 #47	512.5	433.2	6.8	0.410	0.008	1467	12	0.4538	0.0032	1.2474	0.0029	48.55	0.45	1.2839	0.0032
SC-06-01 3 #04	520.5	332.3	3.0	1.413	0.013	326	2	0.4539	0.0025	1.2411	0.0023	48.81	0.36	1.2770	0.0025
SC-06-01 3 #44	523.0	490.1	2.7	0.196	0.002	3382	30	0.4423	0.0020	1.2578	0.0019	46.55	0.27	1.2941	0.0021
SC-06-01 3 #21	531.9	325.2	1.0	0.145	0.001	3024	24	0.4419	0.0023	1.2665	0.0021	46.08	0.310	1.3036	0.0023
SC-06-01 3 #22	535.3	322.2	1.0	0.045	0.001	9768	86	0.4419	0.0021	1.2620	0.0020	46.30	0.29	1.2986	0.0022
SC-06-01 3 #24	537.3	339.6	1.6	0.036	0.001	11940	117	0.4151	0.0016	1.2566	0.0020	43.14	0.22	1.2898	0.0022
SC-06-01 3 #07	538.8	267.7	2.0	0.117	0.001	2877	22	0.4115	0.0024	1.2519	0.0023	42.89	0.32	1.2844	0.0026
SC-06-01 3 #25	539.8	280.0	1.4	0.141	0.002	2504	18	0.4137	0.0017	1.2531	0.0021	43.11	0.23	1.2859	0.0023
SC-06-01 3 #26	541.6	215.9	1.0	0.269	0.003	1013	6	0.4125	0.0018	1.2532	0.0021	42.94	0.24	1.2860	0.0023
SC-06-01 3 #05	545.9	198.5	1.8	0.315	0.004	7889	7	0.4097	0.0036	1.2414	0.0035	43.08	0.48	1.2728	0.0038
SC-06-01 3 #27	546.3	195.9	0.9	0.120	0.002	2044	21	0.4096	0.0023	1.2560	0.0025	42.47	0.31	1.2887	0.0027
SC-06-01 3 #28	549.3	241.6	0.7	0.181	0.001	1626	9	0.3985	0.0017	1.2460	0.0019	41.49	0.23	1.2767	0.0021
SC-06-01 3 #06	551.8	264.2	2.6	0.317	0.003	1003	8	0.3940	0.0026	1.2386	0.0030	41.23	0.36	1.2682	0.0033
SC-06-01 3 #29	551.8	201.0	0.7	0.122	0.001	1964	15	0.3915	0.0020	1.2415	0.0021	40.82	0.26	1.2711	0.0023
SC-06-01 3 #30	554.1	134.0	0.4	0.319	0.002	506	4	0.3938	0.0021	1.2478	0.0021	40.80	0.28	1.2782	0.0023

SC-06-01 3 #31	558.1	302.6	0.8	0.124	0.001	2710	19	0.3629	0.0016	1.2531	0.0020	36.86	0.21	1.2810	0.0022
SC-06-01 3 #08	558.2	363.5	3.6	0.356	0.004	1134	7	0.3635	0.0020	1.2479	0.0024	37.13	0.26	1.2754	0.0027
SC-06-01 3 #32	558.2	409.8	1.2	0.209	0.002	2183	17	0.3640	0.0018	1.2547	0.0020	36.94	0.23	1.2828	0.0021
SC-06-01 3 #33	559.8	425.9	1.0	0.023	0.001	20722	172	0.3595	0.0017	1.2415	0.0020	36.90	0.22	1.2680	0.0022
SC-06-01 3 #01	560.6	343.0	2.7	0.173	0.002	2206	24	0.3631	0.0024	1.2404	0.0027	37.36	0.31	1.2672	0.0029
SC-06-01 3 #34	561.4	404.9	140.9	0.015	0.008	30242	557	0.3635	0.0015	1.2446	0.0022	37.26	0.20	1.2718	0.0024
SC-06-01 3 #48	566.8	320.2	2.6	0.209	0.003	1711	16	0.3649	0.0022	1.2470	0.0025	37.34	0.29	1.2745	0.0027
SC-06-01 3 #45	585.3	323.2	2.1	0.064	0.001	5615	57	0.3611	0.0020	1.2447	0.0021	36.97	0.25	1.2716	0.0023
SC-06-01 3 #46	631.4	227.5	1.5	0.114	0.002	2199	21	0.3599	0.0024	1.2476	0.0026	36.71	0.30	1.2747	0.0028
SC-06-01 3 #47	651.8	262.9	2.1	0.408	0.004	706	6	0.3587	0.0023	1.2510	0.0027	36.42	0.29	1.2784	0.0030
SC-06-01 3 #18	683.6	441.4	2.9	0.045	0.001	10730	146	0.3583	0.0015	1.2481	0.0025	36.52	0.20	1.2750	0.0027
SC-06-01 3 #35	724.5	280.7	0.9	0.080	0.001	3827	35	0.3561	0.0021	1.2497	0.0022	36.19	0.26	1.2766	0.0024
SC-06-01 3 #15	760.2	444.4	3.9	0.717	0.009	665	5	0.3515	0.0020	1.2428	0.0030	35.85	0.26	1.2688	0.0032
SC-06-01 3 #10	822.4	156.0	11.2	0.089	0.011	1872	79	0.3482	0.0033	1.2473	0.0049	35.33	0.43	1.2733	0.0053
SC-06-01 3 #19	862.9	331.6	2.5	0.033	0.001	10345	149	0.3369	0.0019	1.2159	0.0027	35.07	0.25	1.2384	0.0029
SC-06-01 3 #11	896.6	342.0	25.9	0.070	0.012	4977	338	0.3352	0.0023	1.2223	0.0032	34.64	0.30	1.2452	0.0035
SC-06-01 3 #16	908.7	280.4	2.6	0.104	0.002	2765	31	0.3346	0.0026	1.2439	0.0034	33.85	0.32	1.2685	0.0037
SC-06-01 3 #42	913.5	263.3	2.7	0.642	0.009	418	3	0.3334	0.0017	1.2301	0.0022	34.11	0.22	1.2535	0.0024
SC-06-01 3 #17	924.6	252.0	2.3	0.211	0.003	1162	12	0.3182	0.0025	1.2357	0.0034	32.18	0.31	1.2582	0.0037
SC-06-01 3 #43	930.4	288.7	3.3	1.163	0.015	241	2	0.3180	0.0020	1.2342	0.0026	32.13	0.25	1.2567	0.0028
SC-06-01 3 #02	933.6	239.2	1.5	0.224	0.003	1011	11	0.3099	0.0023	1.2087	0.0032	32.04	0.29	1.2286	0.0034
SC-06-01 3 #09	243.6	357.8	26.9	0.038	0.008	9692	930	0.3342	0.0024	1.2150	0.0030	34.78	0.30	1.2372	0.0033

SC-06-01 3 #12	211.3	263.9	21.0	0.091	0.011	2929	117	0.3321	0.0020	1.2100	0.0033	34.69	0.27	1.2317	0.0036
SC-06-01 3 #13	154.3	338.3	26.3	0.160	0.017	2155	71	0.3331	0.0022	1.2236	0.0029	34.34	0.29	1.2464	0.0031
SC-06-01 3 #41	77.3	286.6	3.9	1.063	0.017	274	2	0.3329	0.0024	1.2376	0.0027	33.77	0.30	1.2617	0.0029
SC-06-01 3 #40	58.6	239.1	2.5	0.517	0.007	466	4	0.3295	0.0019	1.2346	0.0029	33.51	0.24	1.2580	0.0032
SC-06-01 3 #36	54.4	212.5	3.2	0.894	0.016	245	3	0.3370	0.0034	1.2249	0.0080	34.67	0.50	1.2483	0.0087
SC-06-01 3 #14	51.1	258.6	2.8	0.138	0.002	1798	23	0.3135	0.0026	1.2284	0.0041	31.86	0.33	1.2499	0.0044
SC-06-01 3 #39	46.3	286.9	4.0	0.492	0.007	566	5	0.3173	0.0019	1.2299	0.0024	32.23	0.23	1.2520	0.0026
SC-06-01 3 #37	37.4	251.7	3.8	0.251	0.013	958	21	0.3129	0.0038	1.2239	0.0034	31.92	0.46	1.2451	0.0036
SC-06-01 3 #38	33.3	756.1	29.0	0.105	0.009	6767	155	0.3068	0.0017	1.2352	0.0025	30.89	0.21	1.2567	0.0026
SC-06-01 3 #20	10.9	402.0	3.2	0.066	0.001	5693	67	0.3065	0.0016	1.2372	0.0028	30.79	0.20	1.2587	0.0030
SC-06-01 3 #03	7.8	348.0	2.1	0.127	0.002	2573	26	0.3064	0.0019	1.2354	0.0026	30.83	0.23	1.2569	0.0028

All ratios are activity ratios. Analytical errors are at 95 % confidence level. Shown are uncorrected activity ratios.

Age calculation is based on $\left(\frac{^{230}Th}{^{238}U}\right)(T) = (1-e^{-\lambda_{230} T}) + \left(\left(\frac{^{234}U}{^{238}U}\right)(T)-1\right)\frac{\lambda_{230}}{\lambda_{230}-\lambda_{234}}(1-e^{-(\lambda_{230}-\lambda_{234})\, T})$ where T is the age of the sample.

The degree of detrital ^{230}Th contamination is indicated by the measured ^{230}Th/^{232}Th activity ratio and corrections were calculated using a ^{238}U/^{232}Th activity ratio of 0.8 ± 0.4.

^{234}U/^{238}U$_{ini}$ is the initial ^{234}U/^{238}U activity ratio.

Ages are calculated based on corrected activity ratios. For all samples, correction is less than 0.1 ka and corrected and uncorrected results are identical within uncertainties.

References

1 Abdulsamad, E. O. Stratigraphy and palaeobiogeography of Tertiary larger foraminifera from Al Jabal al Akhdar (Cyrenaica, NE Libya). *Giornale di Geologia (Bologna)* **61**, 75-98 (1999).

2 Engelstaedter, S., Tegen, I. & Washington, R. North African dust emissions and transport. *Earth-Sci. Rev.* **79**, 73-100, doi:10.1016/j.earscirev.2006.06.004 (2006).

3 Hoffmann, D. L. Th-230 isotope measurements of femtogram quantities for U-series dating using multi ion counting (MIC) MC-ICPMS. *Int. J. Mass Spectrom.* **275**, 75-79, doi:10.1016/j.ijms.2008.05.033 (2008).

4 Hoffmann, D. L. *et al.* Procedures for accurate U and Th isotope measurements by high precision MC-ICPMS. *Int. J. Mass Spectrom.* **264**, 97-109 (2007).

5 Jaffey, A. H., Flynn, K. F., Glendenin, L. E., Bentley, W. C. & Essling, A. M. Precision Measurement of Half-Lives and Specific Activities of U-235 and U-238. *Phys. Rev. C* **4**, 1889-& (1971).

6 Cheng, H. *et al.* The half-lives of uranium-234 and thorium-230. *Chem. Geol.* **169**, 17-33 (2000).

7 Holden, N. E. Total Half-Lives for Selected Nuclides. *Pure Appl. Chem.* **62**, 941-958 (1990).

8 Wedepohl, K. H. The Composition of the Continental-Crust. *Geochim. Cosmochim. Acta* **59**, 1217-1232 (1995).

9 Scholz, D. & Hoffmann, D. L. StalAge - An algorithm designed for construction of speleothem age models. *Quat. Geochronol.* **6**, 369-382, doi:10.1016/j.quageo.2011.02.002 (2011).

10 Svensson, A. *et al.* A 60 000 year Greenland stratigraphic ice core chronology. *Clim. Past.* **4**, 47-57 (2008).

11 Wolff, E. W., Chappellaz, J., Blunier, T., Rasmussen, S. O. & Svensson, A. Millennial-scale variability during the last glacial: The ice core record. *Quat. Sci. Rev.* **29**, 2828-2838, doi:10.1016/j.quascirev.2009.10.013 (2010).

12 Berger, A. & Loutre, M. F. Insolation values for the climate of the last 10000000 years. *Quat. Sci. Rev.* **10**, 297-317, doi:10.1016/0277-3791(91)90033-q (1991).
